# Association of Common Variants in *TNFRSF13B*, *TNFSF13*, and *ANXA3* with Serum Levels of Non-Albumin Protein and Immunoglobulin Isotypes in Japanese

**DOI:** 10.1371/journal.pone.0032683

**Published:** 2012-04-27

**Authors:** Wael Osman, Yukinori Okada, Yoichiro Kamatani, Michiaki Kubo, Koichi Matsuda, Yusuke Nakamura

**Affiliations:** 1 Laboratory of Molecular Medicine, Institute of Medical Science, The University of Tokyo, Tokyo, Japan; 2 Laboratory for Statistical Analysis, Center for Genomic Medicine, Institute of Physical and Chemical Research (Center for Genomic Medicine, RIKEN), Kanagawa, Japan; 3 Department of Allergy and Rheumatology, Graduate School of Medicine, The University of Tokyo, Tokyo, Japan; 4 Centre d’Etude du Polymorphisme Humain, Paris, France; 5 Laboratory for Genotyping Development, Center for Genomic Medicine, RIKEN, Kanagawa, Japan; University of South Florida College of Medicine, United States of America

## Abstract

We performed a genome-wide association study (GWAS) on levels of serum total protein (TP), albumin (ALB), and non-albumin protein (NAP). We analyzed SNPs on autosomal chromosomes using data from 9,103 Japanese individuals, followed by a replication study of 1,600 additional individuals. We confirmed the previously- reported association of *GCKR* on chromosome 2p23.3 with serum ALB (rs1260326, *P*_meta_ = 3.1×10^−9^), and additionally identified the significant genome-wide association of rs4985726 in *TNFRSF13B* on 17p11.2 with both TP and NAP (*P*_meta_ = 1.2×10^−14^ and 7.1×10^−24^, respectively). For NAP, rs3803800 and rs11552708 in *TNFSF13* on 17p13.1 (*P*_meta_ = 7.2×10^−15^ and 7.5×10^−10^, respectively) as well as rs10007186 on 4q21.2 near *ANXA3* (*P*_meta_ = 1.3×10^−9^) also indicated significant associations. Interestingly, *TNFRSF13B* and *TNFSF13* encode a tumor necrosis factor (TNF) receptor and its ligand, which together constitute an important receptor-ligand axis for B-cell homeostasis and immunoglobulin production. Furthermore, three SNPs, rs4985726, rs3803800, and rs11552708 in *TNFRSF13B* and *TNFSF13,* were indicated to be associated with serum levels of IgG (*P*<2.3×10^−3^) and IgM (*P*<0.018), while rs3803800 and rs11552708 were associated with IgA (*P*<0.013). Rs10007186 in 4q21.2 was associated with serum levels of IgA (*P* = 0.036), IgM (*P* = 0.019), and IgE (*P* = 4.9×10^−4^). Our results should add interesting knowledge about the regulation of major serum components.

## Introduction

Serum proteins possess various biological functions such as hormones, enzymes, antibodies, and clotting agents, and some serve as valuable biomarkers that reflect several disease conditions. Major components of serum proteins are ALB (approximately 60%), globulins (mainly as γ-globulins, approximately 30%), and fibrinogens. Total serum protein levels range from 6.5 to 8.5 g/dl and show significant inter-individual variation. These variations are found to be influenced by environmental factors. However, genetic factors are also known to affect their levels although the range of genetic effects varies by the reports from 20% to 77% [1]. Genome-wide association studies (GWAS) recently demonstrated that serum levels of several proteins can be strongly influenced by common genetic variants through either *cis* or *trans* effects [2]–[4].

We previously reported the GWAS results for hematological and biochemical traits, including TP and ALB, in the Japanese population [5]. An associated SNP for TP, rs4273077 (*P*-value = 4.5×10^−10^), is located in an intron of *TNFRSF13B* (Tumor Necrosis Factor Receptor Superfamily member 13B), which encodes TACI (transmembrane activator and calcium-modulator and cytophilin interactor), one of three TNF-receptor family members (BAFF-R, TACI, and BCMA) [6]. However, since rs4273077 showed no significant association with the serum ALB level (*P* = 0.089), we suspected that this SNP would have genetic effects primarily on the levels of the non-albumin fraction. TACI is expressed mainly in activated B cells and binds with a high affinity to two TNF ligands; APRIL (a Proliferation-Inducing Ligand, encoded by *TNFSF13*), and BAFF (B Cell-Activating Factor, encoded by *TNFSF13B*) [7]. TACI is implicated in B- cell homeostasis (including B- cell survival, activation, and differentiation), immunoglobulin production, and antibody class switching [8]–[10]. Hence, the association of variants in *TNFRSF13B* with TP is likely to reflect the immunoglobulin serum levels.

The aim of this study is to identify the genetic variations associated with serum levels of non-albumin proteins (NAP), particularly those of immunoglobulins by GWAS of Japanese subjects.

## Results

### GWAS of Total Protein (TP), Albumin (ALB), and Non-albumin Protein (NAP)

We conducted a GWAS using genotyping data and clinical information on 9,103 individuals who had been collected in the BioBank Japan Project [11] (Table 1, Table S1). Genotyping was performed using Illumina Human610-Quad BeadChip (Illumina, CA, USA). After applying stringent quality control (QC) filters for selection of individuals and SNPs (Materials and Methods), we additionally performed whole-genome imputation analysis using the data of HapMap Phase II East Asian populations, and we obtained the information of 2,178,644 SNPs on autosomal chromosomes with minor allele frequencies (MAF) of ≥0.01 and *Rsq* of ≥0.7. We then evaluated the association of the SNPs with the adjusted *Z* scores of serum levels of total protein (TP), albumin (ALB), and non-albumin protein (NAP). A Quantile-quantile (Q-Q) plot for each trait indicated low possibility of population stratification (inflation factors (λ_GC_) for TP, ALB and NAP were 1.04, 1.02 and 1.02, respectively) (Figure S2).

**Table 1:** Characteristics of the examined proteins.

	TP		ALB		NAP		IgG *	IgA *	IgM *	IgE *
	GWAS	Replication	GWAS	Replication	GWAS	Replication				
No.	9,090	1,626	9,103	1,607	9,077	1,629	1,794	1,675	1,649	549
M±S.D a	7.10±0.50	7.06±0.73	4.25±0.35	4.00±0.51	2.85±0.42	3.07±0.57	1.44±0.61	0.27±0.15	0.11±0.07	1306.54±5598.06
Age b	69.52±10.44	59.52±15.43	69.52±10.44	59.54±15.39	69.51±10.44	59.48±15.52	59.70±15.46	59.38±15.73	59.42±15.57	62.54±18.61
Female %	37.45	45.08	37.41	45.12	37.46	45.12	55.30	54.57	54.88	63.93
BMI b	22.91±3.45	23.31±5.67	22.91±3.45	23.34±5.69	22.91±3.45	23.29±5.67	23.17±5.00	23.20±5.09	23.19±5.07	22.73±4.22
Smokers %	42.11	51.91	42.11	52.15	42.05	51.81	51.90	51.82	52.27	48.63
Drinkers %	29.37	51.97	29.37	52.08	29.40	51.81	51.00	50.81	50.82	41.35

a M±S.D: mean value±standard deviation of each protein is indicated in g/dl except for IgE, which is indicated as IU/ml.

b Age and body mass index (BMI) are indicated as mean values±standard deviation.

* Log-transformed values were applied in the analysis.

Abbreviations: GWAS: genome-wide association study, TP: total protein, ALB: albumin, NAP: non-albumin protein.

**Figure 1:**
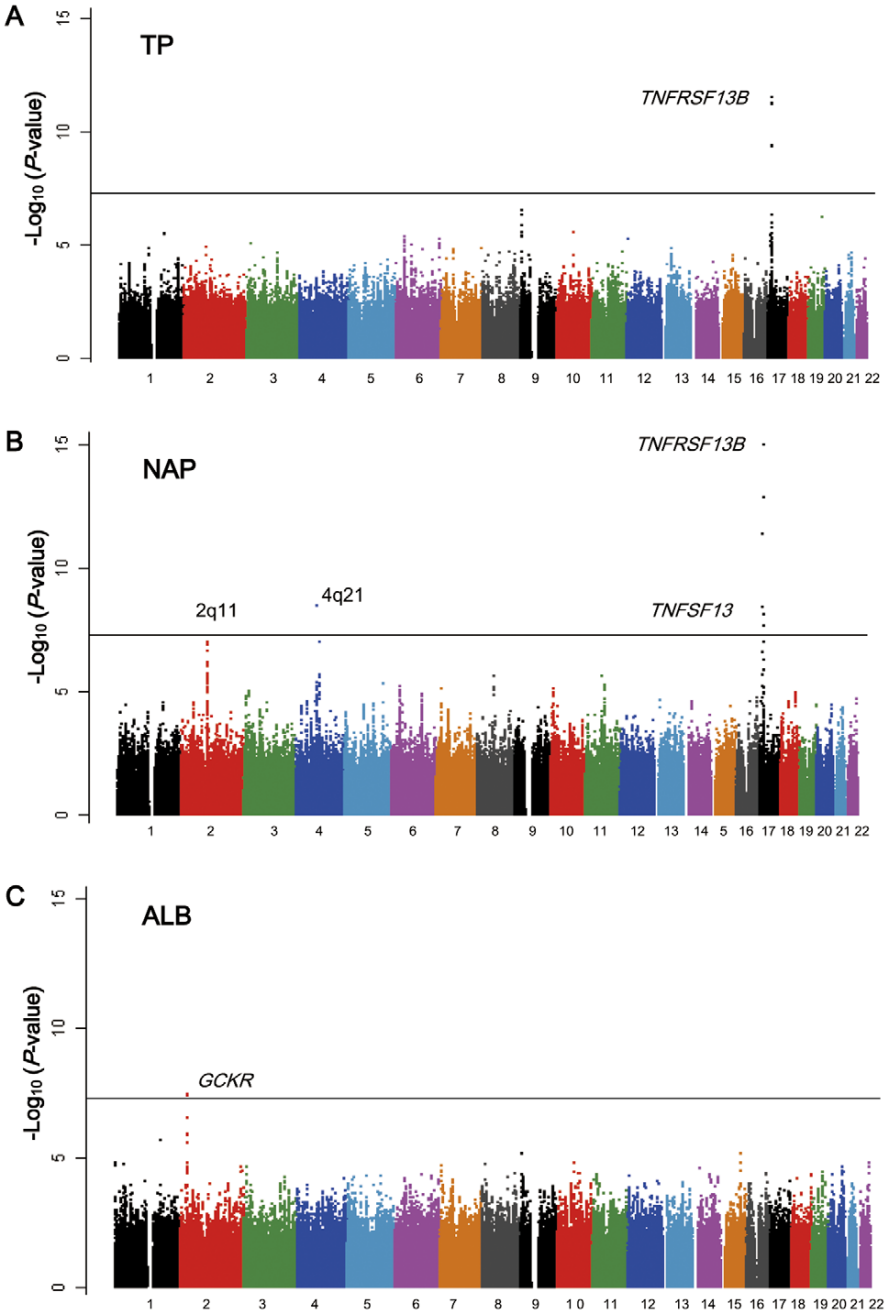
Manhattan plots for the GWAS of (A) TP, (B) NAP, and (C) ALB. SNPs were plotted based on their physical chromosomal positions (horizontal axis) together with their –log_10_ (*P-*values) in the GWAS (vertical axis). The black horizontal line shows the genome-wide significance threshold of *P* = 5.0×10^−8^. The SNPs for which *P*-values were smaller than 1.0×10^−15^ are indicated at the upper limit of the plots.

**Figure 2:**
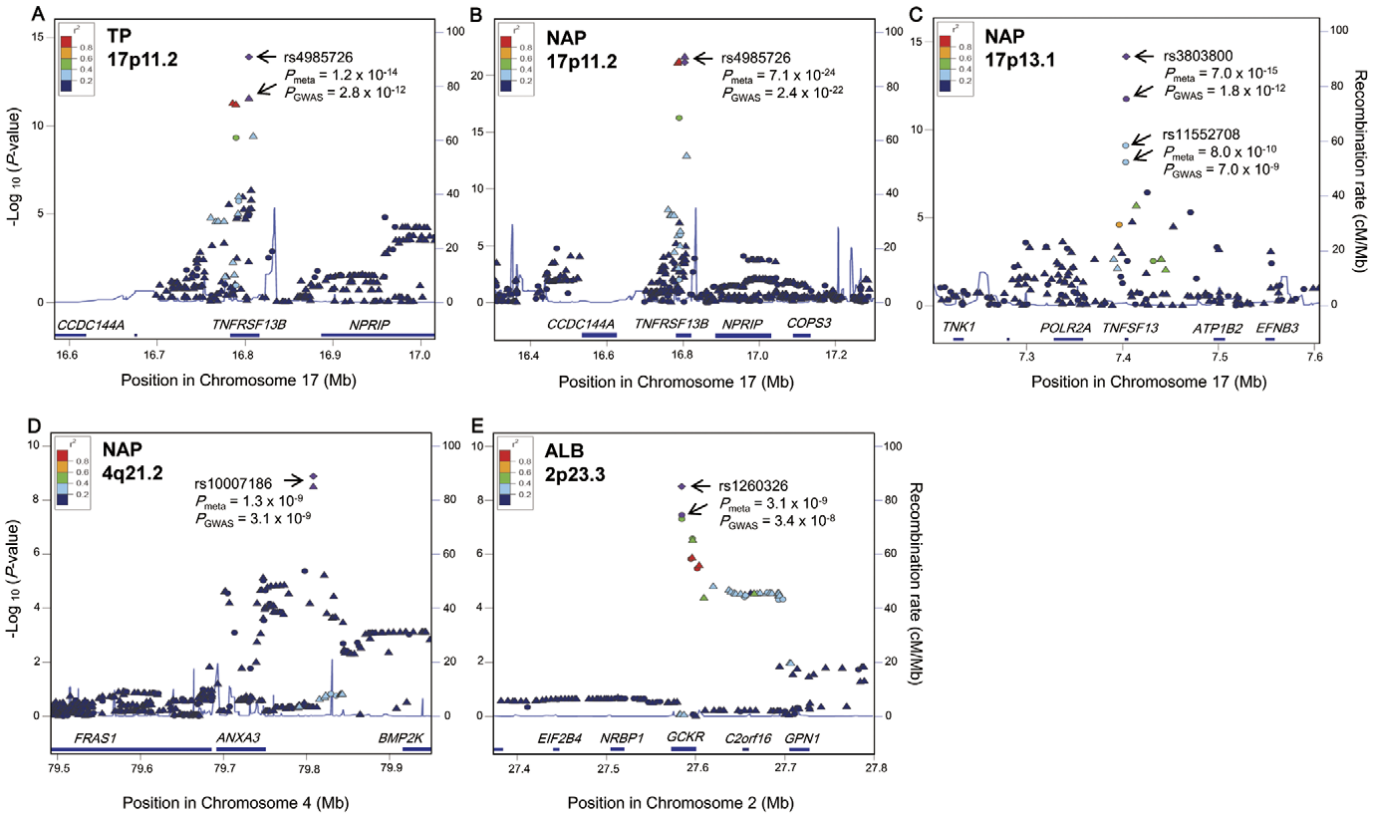
Regional plots for the associations of the SNPs in the GWAS stage of TP, ALB and NAP. SNPs plotted with their –log_10_ (*P*-values) in the GWAS based on their physical chromosomal positions. Genotyped SNPs are indicated as circles, while imputed SNPs are indicated as triangles. The color scheme indicated the linkage disequilibrium displayed as *r* ^2^ values between all SNPs and the top-ranked SNP in each plot. The tested trait, chromosomal locus, and the top-ranked SNPs (in purple color) in the GWAS and combined analyses together with their *P*-values are shown in each plot. The blue lines represent the recombination rates estimated based on HapMap Phase ΙΙ database. The plots were drawn using Locus Zoom software.

Several SNPs with strong linkage disequilibrium (LD) (*r* ^2^>0.8) in intronic regions of *TNFRSF13B* on chromosome 17p11.2 showed significant associations with both TP and NAP (rs4985726, *P* = 2.8×10^−12^ and 2.4×10^−22^, respectively) (Table 2, Table S2, Figure 1A and 1B, and Figure 2A and 2B). In addition, rs3803800 and rs11552708 in coding regions of *TNFSF13* on chromosome 17p13.1 demonstrated significant associations with NAP (*P* = 1.8×10^−12^ and 7.0×10^−9^, respectively) (Table 2, Figure 1B, and Figure 2C).

**Table 2:** Summary results of the GWAS and the replication study of TP, ALB, and NAP.

Trait	SNP	Chr: Position	Nearest Gene	A1/A2^a^	MAF	GWAS		Replication		Meta analysis		% variance explained
						Effect^b^ (s.e)	*P* c	Effect^b^ (s.e)	*P* c	Effect^b^ (s.e)	*P* c	
**TP**	rs4985726*	17∶16804363	*TNFRSF13B*	C/G	0.375	0.108 (0.015)	2.8×10^−12^	0.100 (0.030)	0.0010	0.107 (0.0138)	1.2×10^−14^	0.53
**ALB**	rs1260326	2∶27584444	*GCKR*	T/C	0.445	−0.082 (0.015)	3.4×10^−8^	−0.070 (0.032)	0.029	−0.080 (0.014)	3.1×10^−9^	0.32
**NAP**	rs4985726*	17∶16804363	*TNFRSF13B*	C/G	0.375	0.148 (0.015)	2.4×10^−22^	0.090 (0.028)	0.0013	0.135 (0.013)	7.1×10^−24^	1.03
	rs3803800	17∶7403693	*TNFSF13*	G/A	0.311	0.108 (0.015)	1.8×10^−12^	0.090 (0.029)	0.0022	0.104 (0.013)	7.2×10^−15^	0.53
	rs11552708	17∶7403279	*TNFSF13*	G/A	0.401	−0.084 (0.015)	7.0×10^−9^	−0.070 (0.027)	0.0091	−0.081 (0.013)	7.5×10^−10^	0.36
	rs10007186*	4∶79808069	*ANXA3*	T/C	0.307	0.095 (0.016)	3.3×10^−9^	0.053 (0.029)	0.065	0.085 (0.014)	1.3×10^−9^	0.38

a A1/A2: major/minor alleles.

b The effect of the minor allele on the normalized values based on an additive genetic model.

c For the GWAS and replication analysis, *P*-values were obtained by linear regression test model, for the Meta analysis by inverse-variance method.

* SNPs obtained by whole-genome imputation analysis.

Abbreviations: GWAS: genome-wide association study, MAF: minor allele frequency, TP: total protein, ALB: albumin, NAP: non-albumin protein, s.e: standard error.

Since *TNFSF13* encodes APRIL, a ligand of TACI encoded by *TNFRSF13B*, this ligand- receptor interaction is likely to play a critical role in regulation of the serum NAP levels. However, we did not find any synergistic effects between SNPs in the receptor and ligand on NAP levels.

Rs10007186 located near *ANXA3* (annexin A3) on chromosome 4q21.2 also revealed significant association with NAP (*P* = 3.3×10^−9^; Table 2, Figure 1B, and Figure 2D), and a cluster of highly linked SNPs near the 5′ flanking region of *AFF3* (AF4/FMR2 family, member 3) on 2q11.2 indicated suggestive associations with NAP (rs4851274, *P* = 9.95×10^−8^) (Table S2). For serum ALB, SNPs rs1260326 (in exon) and rs3817588 (in intron) in *GCKR* (glucokinase regulator) on 2p23.3 revealed significant associations (*P* = 3.4×10^−8^ and 4.1×10^−8^, respectively) (Figure 2E, Table 2, and Table S2).

Conditional logistic regression analysis for the SNPs on 17p13.1 indicated that both rs3803800 and rs11552708 conferred independent associations with NAP levels when adjusted for each other (*P*<0.023). These two SNPs were in strong LD (*D*′ = 0.99, *r* ^2^ = 0.30) and the haplotype analysis of these two SNPs identified that a haplotype (rs3803800 [A] – rs11552708 [G]) revealed stronger association with NAP than individual SNP (*P* = 2.59×10^−13^) (Table S3). Similarly, rs1260326 and rs3817588 in *GCKR* exhibited independent associations with ALB levels (*P*<0.022), and were in LD (D′ = 0.95, *r* ^2^ = 0.50). Moreover, the haplotype (rs1260326 [C]–rs3817588 [C]) indicated stronger association with serum ALB (*P* = 2.83×10^−9^) (Table S4). For the 17p11.2 and 4q21.2 loci, no SNP remained significant after accounting for the effect of marker the SNPs rs4985726, and rs10007186, respectively.

When we examined the genetic contribution of these variations for the traits, the combinations of the SNPs indicated above could explain nearly 0.5%, 2.3%, and 0.3% of variations in serum TP, NAP, and ALB, respectively.

### Replication Study

To validate the GWAS results, we performed a replication study using an independent set of ∼1,600 subjects from BioBank Japan [11] (Table 1). For each trait, we selected marker SNPs for the replication analysis at each locus that indicated the genome-wide significant level of 5.0×10^−8^ (rs4985726 in *TNFRSF13B*, rs3803800 in *TNFSF13*, rs1260326 in *GCKR*, and rs10007186 on 4q21.2). In addition, the two SNPs that remained significant after accounting for the effect of each marker SNP at two loci (rs11552708 in *TNFSF13* and rs3817588 in *GCKR*) were also further investigated.

SNPs rs4985726 in the *TNFRSF13B* locus as well as rs3803800 and rs11552708 in the *TNFSF13* locus revealed significant associations with both TP and NAP (Table 2). The association of rs1260326 in *GCKR* with serum ALB was also replicated (*P* = 0.029; Table 2). Meta-analyses combining the GWAS and the replication study yielded stronger associations of these SNPs than the GWAS alone (Table 2 and Figure 2A, B, C, and E). Rs10007186 near *ANXA3* revealed a suggestive association in the replication study (*P* = 0.065), and meta-analyses indicated that the association was unlikely to be false positive (*P* = 1.3×10^−9^) (Table 2 and Figure 2D).

### Association of the SNPs Identified in the GWAS of NAP with Serum Immunoglobulin Isotypes

Immunoglobulin isotypes constitute the major components of NAP. Hence, we further examined the NAP-associated SNPs in the GWAS (*TNFRSF13B*, *TNFSF13*, and *ANXA3*) for the association with various serum immunoglobulins using the samples in BioBank Japan [11] (IgG: *n* = 1,794, IgA: *n* = 1,675, IgM: *n* = 1,649, and IgE: *n* = 549; Table 1).

We found significant associations of rs4985726 in *TNFRSF13B* as well as rs3803800 and rs11552708 in *TNFSF13* with serum levels of IgG (*P*<0.0023) and IgM(*P*<0.018) (Table 3). For IgA, rs3803800 and rs11552708 in *TNFSF13* also revealed the significant association (*P*<0.013), while rs4985726 in *TNFRSF13B* revealed no significant association (*P* = 0.099) (Table 3). Rs10007186 near *ANXA3* indicated significant association with IgA (*P* = 0.036), IgM (*P* = 0.019), and IgE (*P* = 4.9×10^−4^). However, these associated SNPs explained only 1.4%, 0.9%, 1.3%, and 2.0% of the variances of log-transformed values of serum IgG, IgA, IgM, and IgE, respectively.

**Table 3:** Association of the SNPs in the GWAS of the NAP with immunoglobulin isotypes.

SNP	Gene	IgG			IgA			IgM			IgE		
		Effect a (s.e)	*P* b	%EV	Effect a (s.e)	*P* b	%EV	Effect a (s.e)	*P* b	%EV	Effect a (s.e)	*P* b	*%E*V
rs4985726	*TNFRSF13B*	0.071 (0.022)	1.4×10^−3^	0.51	0.049 (0.030)	0.099	–	−0.090 (0.032)	5.9×10^−3^	0.40	0.039 (0.064)	0.54	–
rs3803800	*TNFSF13*	−0.074 (0.024)	2.2×10^−3^	0.47	−0.086 (0.031)	6.2×10^−3^	0.39	−0.082 (0.034)	0.018	0.29	−0.117 (0.067)	0.080	–
rs11552708	*TNFSF13*	0.067 (0.022)	2.3×10^−3^	0.46	0.072 (0.029)	0.013	0.31	0.078 (0.032)	0.014	0.31	0.059 (0.060)	0.33	–
rs10007186	*ANXA3*	−0.018 (0.022)	0.42	–	−0.063 (0.030)	0.036	0.20	−0.078 (0.033)	0.019	0.27	0.200 (0.057)	4.9×10^−4^	2.02

a The effect of the minor alleles on the standardized values.

b *P*-values for the associations of SNPs with each normalized immunoglobulin isotype obtained by using a linear regression model.

Abbreviations: s.e: standard error, %EV: percentage of the explanatory variance.

## Discussion

On the basis of the information of 10,716 Japanese individuals, we identified one genetic locus (*TNFRSF13B*) on chromosome 17p11.2 associated with both TP and NAP, two loci (*TNFSF13* on 17p13.1 and a region near *ANXA3* on 4q21.2) associated with NAP, and one locus (*GCKR*) on 2p23.3 associated with ALB at the level of genome-wide significance.

The marker SNP rs4985726 shows association with TP and NAP is located in an intron of *TNFRSF13B* on chromosome 17p11.2. A possible mechanism for its association with these traits could be explained by its strong LD with rs34562254 (*D*′ = 1, *r* ^2^ = 0.97), which exhibits a missense variation (C>T, Pro251Leu) located in the intracellular domain of the receptor molecule. The *in silico* prediction of the amino acid substitution by rs34562254 in the PolyPhen-2 and SNPinfo database [12], [13] suggested a “probably damaging” effect on the protein structure.

The SNPs in *TNFSF13* (encoding APRIL) that identified as being associated with NAP are missense variants; rs3803800 (A>G, Asn96Ser), and rs11552708 (G>A, Gly67Arg). APRIL was first described as having a promoter function for tumor-cell proliferation and survival [14]. APRIL is cleaved in the Golgi apparatus by furin at its 104Arg/105Ala site [15], and interestingly, rs3803800 is closely located to this cleavage site. Hence, this SNP might affect the cleavage affinity. Another possibility is the effect on splicing, because both SNPs are predicted to be located within binding sites of splicing regulatory elements [13]. However, further investigation should be required to address these possibilities.

The SNP rs4985726 in *TNFRSF13B* as well as rs3803800 and rs11552708, in *TNFSF13* also revealed significant associations with serum levels of IgG, IgA, and IgM. It is notable that the two genes encode a TNF-receptor and ligand axis that plays important roles for mediating antibody class switching and regulating immunoglobulin production [8], [9]. Furthermore, knockout mice of either *TNFRSF13B* or *TNFSF13* presented a common phenotype of the IgA deficiency with impaired antibody response to T cell-independent antigens [16]. In addition, germ-line mutations in *TNFRSF13B* were reported in cases of common variable immunodeficiency (CVID; MIM # 607594) and selective IgA deficiency (IGAD; MIM # 137100) [17]. The combination of these significant statistical and biological evidences would suggest that the association of these two loci with NAP reflect at least their associations with regulation of serum immunoglobulin levels. It is also known that immunoglobulins are the major components of NAP, which provides compelling evidence for our results. The facts that both SNPs rs3803800 [A] and rs11552708 [G] in *TNFSF13* were reported to be associated with the susceptibility to the Systemic Lupus Erythematosus (SLE) in the Japanese population and that high serum APRIL was detected in the sera of individuals with the rs3803800 [A]–rs11552708 [G] haplotype [18] further support the significance of these SNPs in the regulation of immunoglobulin production. In this study, we observed that possession of two copies of SLE-risk alleles was associated with higher serum levels of NAP, IgG, IgA, and IgM (Figure S3), providing a good example of genetic loci that influence both quantitative traits and susceptibility to complex diseases.

Rs10007186, which was associated with NAP (*P*_meta_ = 1.3×10^−9^) is located about 57.4 kb downstream of *ANXA3* encoding annexin A3, a member of annexin family of calcium-dependent phospholipid-binding proteins [19]. Annexin A3 was found to be translocated into phagosomes in dendritic cells [20], which are antigen-presenting cells that serve as messengers between the innate and adaptive immune response, and play a key role in allergic, inflammatory, and autoimmune conditions. In addition, annexin A3 was also found to be associated with neutrophil granule membranes [21], where it can play a regulatory role in calcium-dependent granule secretions that contribute to acute inflammation and chronic tissue destruction. The association of rs10007186 with IgA, IgM, and IgE, would suggest additional biological roles of annexin A3 in the immune response.

We also confirmed the association of SNPs in *GCKR* with serum ALB levels (rs1260326, *P*_meta_ = 3.1×10^−9^). Rs1260326 is a missense variant (T>C, Leu446Pro) and predicted to cause a damaging effect on the protein structure. *GCKR* is a locus frequently associated with several metabolic traits [4], [22]–[24] and rs1260326 has been reported to be associated with serum triglycerides [4].

As a conclusion, the present study identified genetic loci that influence the inter-individual variation in serum levels of TP, ALB, and NAP. The loci associated with NAP encompass genes encoding a TNF-receptor and its ligand, which are implicated in biological roles in the immune system, and their associations with immunoglobulin isotypes were demonstrated here. Our results should add novel insight toward understanding the genetic background contributing to the regulation of the serum levels of NAP and its major components.

## Materials and Methods

### Study Cohorts

For the GWAS, 9,103 subjects derived from 10 disease cohorts (colorectal cancer, breast cancer, prostate cancer, lung cancer, gastric cancer, diabetes mellitus, peripheral artery disease, atrial fibrillation, ischemic stroke, and myocardial infarction) were selected, and for the replication study, we used data from >1600 independent individuals selected from the BioBank Japan Project [11] (Table 1 and Table S1). For immunoglobulin isotypes analyses, the data from ∼1,600 additional individuals in BioBank Japan [11] was used (Table 1). The clinical information for the samples is updated annually using a standard questionnaire in the 66 hospitals participating in the project. Written informed consent was obtained from all subjects. The research project was approved by the ethical committees in the Institute of Medical Science, the University of Tokyo, and the Center of Genomic Medicine, RIKEN, Yokohama, Japan.

### Genotyping and Quality Control (Q.C) Filters

In the GWAS, SNPs were genotyped using the Illumina HumanHap610-Quad BeadChip (Illumina, CA, USA). After the exclusion of samples with call rates of <0.98, we excluded closely related individuals (in 1^st^ or 2^nd^ degree kinships) using identity-by-descent (IBD) evaluated by PLINK version 1.0.6 [25]. We also excluded individuals who were outliers in the cluster analysis using the principle component analysis performed by EIGENSTRAT 3.0 along with HapMap Phase ΙΙ populations (Figure S1). In addition, SNPs with call rates of <0.99, MAF of <0.01 and Hardy Weinberg equilibrium of *P* <1.0×10^−7^ were excluded.

Genotyping data of the SNPs selected for replication analyses and for testing with immunoglobulin levels were generated using multiplex PCR- based Invader Assay (Third Wave Technologies, Madison, WI, USA) [26]. Genotypes were judged by visual inspection, following the application of QC measures of individuals’ call rates of >98% and SNPs call rates of >99% of individuals. We could not obtain the genotype data of rs3817588 in *GCKR* using the Invader assay.

### Whole-genome Imputation of Genotypes

We performed whole-genome imputation of the GWAS subjects in a two-step procedure, as described elsewhere [27]. HapMap phase ΙΙ Japanese (JPT) and Han Chinese (CHB) individuals (release 24) were adopted as reference panels. We excluded the imputed SNPs with MAF of <0.01 or *Rsq* of <0.7. As a result, a total of 2,178,644 SNPs on autosomal chromosomes were used for the GWAS.

### Statistical Analysis

We obtained the non-transformed values of TP, ALB and NAP (mg/dl) for the subjects from the clinical information stored in BioBank Japan [11], and adjusted them in linear regression models with age, gender, body mass index (BMI), smoking, drinking status, and affection status of the disease as covariates. The residuals were then normalized as *Z* scores and subjects with *Z* scores of <−4 or >4 were removed from each trait analysis. The associations of the SNPs with *Z* scores were evaluated in linear regression models assuming additive effects of allele dosages, using mach2qtl software. The same methods of data normalization and statistical models were applied for the replication analyses and for testing the association with common log-transformed values of immunoglobulin isotypes (IgG IgA, IgM, and IgE). Meta-analyses of the GWAS and the replication study were performed using the inverse-variance method assuming a fixed-effects model.

The significance level used was 5×10^−8^ in the GWAS stage. For the replication stage, we considered 0.05 as significant for the association of rs4985726 with TP and rs1260326 with ALB. For the association of SNPs rs4985726 in TNFRSF13B, rs3803800 and rs11552708 in TNFSF13 with NAP, 0.017 (0.05/3) was considered to be significant. These significance levels represent the Bonferroni correction for multiple statistical tests. In addition, we set a level of 0.05 to consider the association of the selected SNPs with immunoglobulin isotypes as significant.

The haplotype analyses were performed using the Haplo Stats package (version 1.4.0) implemented in *R* statistical software. Epistatic effects of the SNPs in *TNFRSF13B* and *TNFSF13* were evaluated using a linear regression model incorporating the product of the allele dosages of the SNPs in the loci as an independent variable. All statistical analyses including haplotype analyses were performed using the *R* statistical software version 2.9.1 except for genome-wide linear regression analyses. LD analyses were performed using Haploview 4.2 software, PLINK, and the SNAP database.

### Web Resources

The URLs for the data presented in this paper are as follows:

The BioBank Japan Project, http://biobankjp.org/

PLIKN software, http://pngu.mgh.harvard.edu/~purcell/plink/

EIGENSTRAT software, http://genepath.med.harvard.edu/~reich/EIGENSTRAT.htm

The International HapMap Project, http://www.hapmap.org/

MACH and mach2qtl software, http://www.sph.umich.edu/csg/abecasis/MaCH/index.html

R statistical environment, http://www.r-project.org/

Haploview software, www.broad.mit.edu/mpg/haploview/

SNAP, http://www.broadinstitute.org/mpg/snap/ldsearch.php

Locus Zoom, http://csg.sph.umich.edu/locuszoom/

## Supporting Information

Figure S1 **Principal component analysis Plot of cohorts included in the GWAS**. All individuals who were finally incorporated in the GWAS together with the four populations in the HapMap Phase ΙΙ database (Japanese: JPT; Han Chinese: CHB; Africans: YRI, and European: CEU) were plotted based on the first two eigenvectors.
(PDF)

Figure S2 **Quantile-Quantile (Q-Q) plots for the GWAS of (A) TP, (B) NAP, and (C) ALB**. The inflation factor, λ_GC_, for the analysis is shown in the legend of each plot. The SNPs for which *P*-values were smaller than 1.0×10^−15^ are indicated at the upper limit of the plots.
(PDF)

Figure S3 **Relationship between the genotypes of SNPs identified in the study and the levels of tested proteins: (A) rs4985726, (B) rs3803800, (C) rs11552708, (D) rs10007186, and (E) rs1260326.** For each box plot, the bold line indicates the median value which is the 50^th^ quartile. The limits of each box are the 25^th^ and 75^th^ quartiles.
(PDF)

Table S1 **Characteristics of the GWAS cohorts.**
(DOC)

Table S2 **SNPs showed suggestive associations with each examined trait (** ***P*** **<1.0**×**10** ^−**6**^ **).**
(DOC)

Table S3 **Haplotype analysis of rs3803800 and rs11552708 in** ***TNFSF13*** **in association with NAP.**
(DOC)

Table S4 **Haplotype analysis of rs1260326 and rs3817588 in** ***GCKR*** **in association with ALB.**
(DOC)
