# Supplementary figures and images for: Association of Common Variants in TNFRSF13B, TNFSF13, and ANXA3 with Serum Levels of Non-Albumin Protein and Immunoglobulin Isotypes in Japanese
Source: PLoS One. 2012 Apr 27;7(4):e32683. doi: 10.1371/journal.pone.0032683 (PMC3338726; doi:10.1371/journal.pone.0032683)

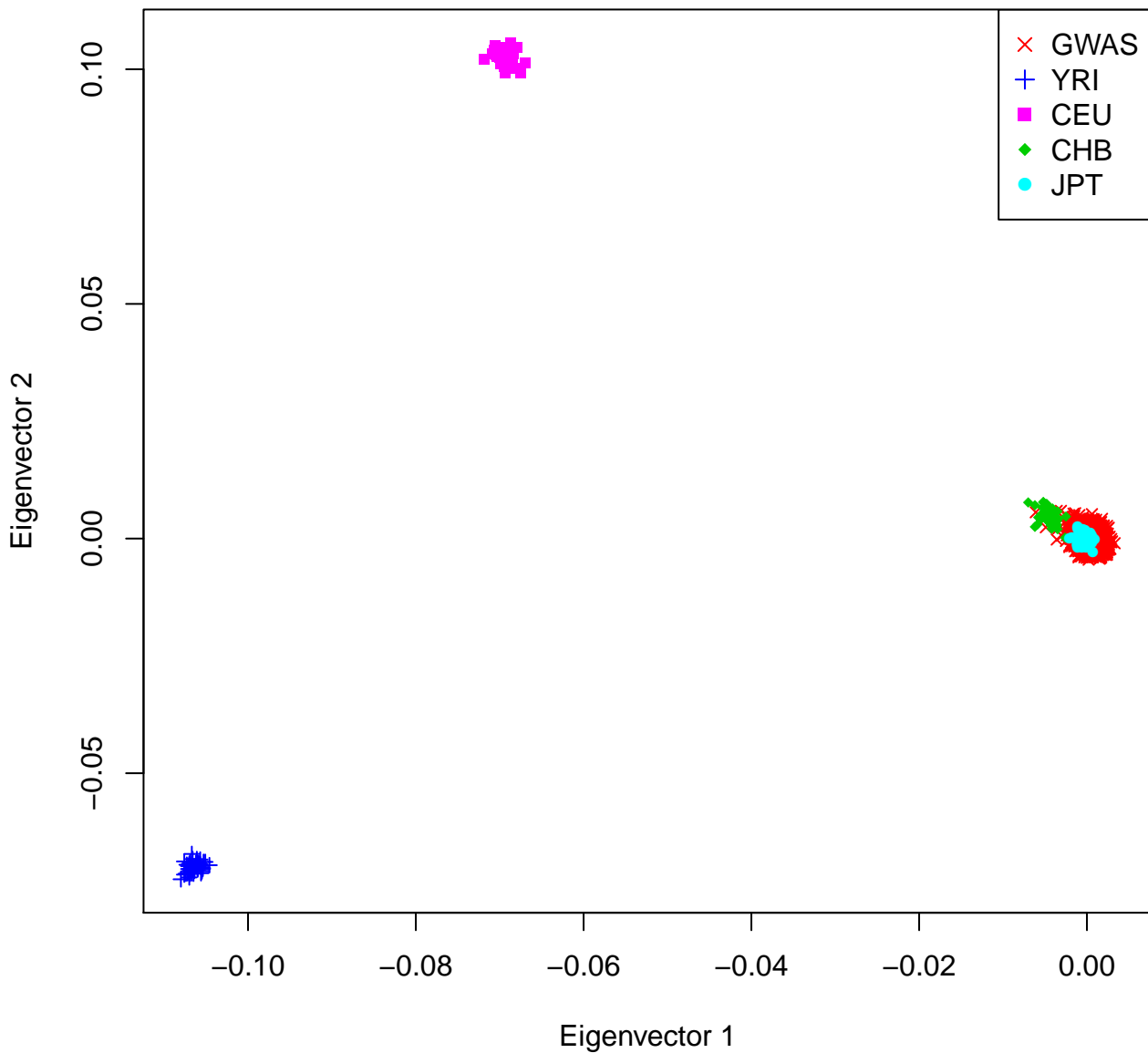

Supplement: Figure S1 — Principal component analysis Plot of cohorts included in the GWAS. All individuals who were finally incorporated in the GWAS together with the four populations in the HapMap Phase ΙΙ database (Japanese: JPT; Han Chinese: CHB; Africans: YRI, and European: CEU) were plotted based on the first two eigenvectors. (PDF) [file pone.0032683.s001.pdf]
