# Supplementary material for: Association of Common Variants in TNFRSF13B, TNFSF13, and ANXA3 with Serum Levels of Non-Albumin Protein and Immunoglobulin Isotypes in Japanese
Source: PLoS One. 2012 Apr 27;7(4):e32683. doi: 10.1371/journal.pone.0032683 (PMC3338726; doi:10.1371/journal.pone.0032683)
Supplement: Figure S2 — Quantile-Quantile (Q-Q) plots for the GWAS of (A) TP, (B) NAP, and (C) ALB. The inflation factor, λGC, for the analysis is shown in the legend of each plot. The SNPs for which P-values were smaller than 1.0×10^−15^ are indicated at the upper limit of the plots. (PDF) [file pone.0032683.s002.pdf]

PLoS Genetics

Novel Associations of TNFRSF13B, TNFSF13, and ANXA3 with Serum levels of Non-albumin Protein and Immunoglobulin Isotypes in the Japanese Population

--Manuscript Draft--

**Manuscript Number:**	
**Full Title:**	Novel Associations of TNFRSF13B, TNFSF13, and ANXA3 with Serum levels of Non-albumin Protein and Immunoglobulin Isotypes in the Japanese Population
**Short Title:**	TNFRSF13B, TNFSF13, and ANXA3 and Serum Proteins
**Article Type:**	Research Article
**Section/Category:**	Natural Variation
**Keywords:**	GWAS; proteins; QTL; TNFRSF13B; TNFSF13; ANXA3
**Corresponding Author:**	Wael Mohammed Osman, M.D<br>Human Genome Center, Institute of Medical Science, the University of Tokyo<br>Tokyo, Tokyo JAPAN
**Corresponding Author Secondary Information:**	
**Corresponding Author's Institution:**	Human Genome Center, Institute of Medical Science, the University of Tokyo
**Corresponding Author's Secondary Institution:**	
**First Author:**	Wael Mohammed Osman, M.D
**First Author Secondary Information:**	
**All Authors:**	Wael Mohammed Osman, M.D
	Yusuke Nakamura
	Yukinori Okada
	Yoichiro Kamatani
	Michiaki Kubo
	Koichi Matsuda
**All Authors Secondary Information:**	
**Abstract:**	We performed a genome-wide association study (GWAS) of serum levels of total protein (TP), albumin (ALB), and non-albumin protein (NAP). We analyzed autosomal SNPs using data from 9,103 Japanese individuals, followed by a replication study of 1,600 additional individuals. We confirmed the previously-reported association of GCKR on 2p23.3 with serum ALB (rs1260326, Pmeta = 3.1x10-9), and newly identified the genome-wide level of significant association of rs4985726 in TNFRSF13B on 17p11.2 with both TP and NAP (Pmeta = 1.2x10-14 and 7.1x10-24, respectively). For NAP, rs3803800 and rs11552708 in TNFSF13 on 17p13.1 (Pmeta = 7.2x10-15 and 7.5x10-10, respectively) as well as rs10007186 on 4q21.2 near ANXA3 (Pmeta = 1.3x10-9) also indicated the significant associations. Interestingly, TNFRSF13B and TNFSF13 encode a tumor necrosis factor (TNF) receptor and its ligand, which constitute an important receptor-ligand axis for B-cell homeostasis and immunoglobulin production. Furthermore, three SNPs, rs4985726, rs3803800, and rs11552708 in TNFRSF13B and TNFSF13, were indicated to be associated with serum levels of IgG (P < 2.3x10-3) and IgM (P < 0.018), while rs3803800 and rs11552708 were associated with IgA (P < 0.013). Rs10007186 on 4q21.2 was associated with those of IgA (P = 0.036), IgM (P = 0.019), and IgE (P = 4.9x10-4). Our results should add interesting knowledge for the regulation of major serum components.
**Suggested Reviewers:**	Christian Gieger<br>German Research Center for Environmental Health (GmbH), Neuherberg, Germany<br>christian.gieger@helmholtz-muenchen.de

Powered by Editorial Manager® and Preprint Manager® from Aries Systems Corporation

	Specialist of QTL-GWAS analyses
	Nicole Soranzo<br>Wellcome Trust Sanger Institute, Hinxton, UK<br>ns6@sanger.ac.uk<br>Specialist of QTL-GWAS analyses.
	Norihiro Kato<br>Research Institute, National Center for Global Health and Medicine, Tokyo, Japan.<br>nokato@ri.ncgm.go.jp<br>Specialist of QTL-GWAS analyses.
	Robert M. Plenge<br>Brigham and Women's Hospital, Harvard Medical School<br>rplenge@partners.org<br>He is an expert in genome-wide association studies for immune-related conditions.
**Opposed Reviewers:**	Nora Franceschini<br>University of North Carolina at Chapel Hill<br>franc016@email.unc.edu<br>Dr. Franc is a member of CHARGE consortium and now doing a meta-analysis of TP and ALB GWAS, which could potentially have conflicts with our study.

Powered by Editorial Manager® and Preprint Manager® from Aries Systems Corporation

Human Genome Center
Institute of Medical Science
The University of Tokyo

4-6-1 Shirokanedai, Minato-ku,
Tokyo 108-8639, Japan
TEL: +81-(0)3-5449-5127
FAX: +81-(0)3-5449-5123

November 25, 2011

To: *PLos Genetics* editorial office
For online submission

Dear editors

Please find the enclosed manuscript entitled "**Novel Associations of *TNFRSF13B*, *TNFSF13*, and *ANXA3* with Serum levels of Non-albumin Protein and Immunoglobulin Isotypes in the Japanese Population**" by Osman et al. which we would like to submit for publication in "*PLos Genetics*" as a research article.

As the most abundant compounds in the serum, proteins possess various biological functions and some of them are important diagnostic markers. The effect of genetic factors on protein levels was found to range from 20% to 77%, and genome-wide association studies (GWAS) revealed their levels can be strongly influenced by common genetic variations. However, to date, no GWAS examining the non-albumin protein fraction of total protein (obtained by subtracting albumin form total protein), in particular those related to immunoglobulins, has been reported.

In this study, we analyzed 10,716 Japanese subjects for 2,178,644 autosomal SNPs using a GWAS and a replication study. We confirmed the association of SNPs in a *GCKR* locus on chromosome 2p23.3 with ALB (P_{meta} = 3.1x10^{-9}), and identified novel cross-trait associations of SNPs in the *TNFRSF13B* locus on 17p11.2 with both total protein (TP) and non-albumin protein (NAP) (P_{meta} = 1.2x10^{-14} and 7.1x10^{-24}, respectively). For NAP, SNPs in the *TNFSF13* locus on 17p13.3 (P_{meta} = 7.2x10^{-15}) and those on 4q21.1 (P_{meta} = 1.3x10^{-9}) also revealed significant associations. *TNFRSF13B* and *TNFSF13* encode a tumor necrosis factor (TNF) receptor and its ligand, respectively, which constitute an important axis for B cells homeostasis and immunoglobulins production. The genetic loci implicated that the association with NAP levels demonstrated significant associations when evaluated with the serum immunoglobulin isotypes.

These results should add useful insight to the understanding of the genetic background contributing to regulation of the serum levels of TP and its major components.

I hope you to find the significance of our manuscript and am looking forward to hearing a positive reply from you soon.

Sincerely,

Yusuke Nakamura, M.D., Ph.D.

Information on the manuscript

Format of the paper: research article

The number of words in the abstract: 212

The number of panels in the paper: 5; 2 figures and 3 tables

Supplementary data: 7; 3 figures, 4 tables, and 0 texts

The number of references: 27

Conflict of interest: none

Postal address:

Human Genome Center
Institute of Medical Science,
The University of Tokyo, 4-6-1,
Shirokanedai, Minato-ku, Tokyo 108-8639, Japan
Phone: +81-3-5449-5372
Fax: +81-3-5449-5433
E-mail: yusuke@ims.u-tokyo.ac.jp

*Manuscript
Click here to download Manuscript: The manuscript_Osman et al..doc

Novel Associations of *TNFRSF13B*, *TNFSF13,* and *ANXA3* with Serum levels of Non-albumin Protein and Immunoglobulin Isotypes in the Japanese Population.

Wael Osman [1] ,Yukinori Okada [2, 3] , Yoichiro Kamatani [4] , Michiaki Kubo [5], Koichi Matsuda [1] & Yusuke Nakamura [1,*]

1- Laboratory of Molecular Medicine, Institute of Medical Science, the University of Tokyo, Tokyo, Japan. 2- Laboratory for Statistical Analysis, Center for Genomic Medicine, Institute of Physical and Chemical Research (CGM, RIKEN), Kanagawa, Japan. 3- Department of Allergy and Rheumatology, Graduate School of Medicine, the University of Tokyo, Tokyo, Japan. 4- Centre d`Etude du Polymorphisme Humain (CEPH), Paris, France. 5- Laboratory for Genotyping Development, CGM, RIKEN, Kanagawa Japan.

[*]Corresponding should be addressed to:

Professor: Yusuke Nakamura, M.D., Ph.D.

Human Genome Center, Institute of Medical Science, University of Tokyo

4-6-1, Shirokanedai, Minato-ku, Tokyo 108-8639, Japan.

Phone: +81-3-5449-5372.

Fax: +81-3-5449-5433.

E-mail: yusuke@ims.u-tokyo.ac.jp

Abstract

We performed a genome-wide association study (GWAS) of serum levels of total protein (TP), albumin (ALB), and non-albumin protein (NAP). We analyzed autosomal SNPs using data from 9,103 Japanese individuals, followed by a replication study of 1,600 additional individuals. We confirmed the previously-reported association of *GCKR* on 2p23.3 with serum ALB (rs1260326, P_{meta} = 3.1x10^{-9}), and newly identified the genome-wide level of significant association of rs4985726 in *TNFRSF13B* on 17p11.2 with both TP and NAP (P_{meta} = 1.2x10^{-14} and 7.1x10^{-24}, respectively). For NAP, rs3803800 and rs11552708 in *TNFSF13* on 17p13.1 (P_{meta} = 7.2x10^{-15} and 7.5x10^{-10}, respectively) as well as rs10007186 on 4q21.2 near *ANXA3* (P_{meta} = 1.3x10^{-9}) also indicated the significant associations. Interestingly, *TNFRSF13B* and *TNFSF13* encode a tumor necrosis factor (TNF) receptor and its ligand, which constitute an important receptor-ligand axis for B-cell homeostasis and immunoglobulin production. Furthermore, three SNPs, rs4985726, rs3803800, and rs11552708 in *TNFRSF13B* and *TNFSF13,* were indicated to be associated with serum levels of IgG (P < 2.3x10^{-3}) and IgM (P < 0.018), while rs3803800 and rs11552708 were associated with IgA (P < 0.013). Rs10007186 on 4q21.2 was associated with those of IgA (P = 0.036), IgM (P = 0.019), and IgE (P = 4.9x10^{-4}). Our results should add interesting knowledge for the regulation of major serum components.

Author Summary

Serum proteins have various biological functions and are valuable as diagnostic markers for many disease conditions. Protein levels in serum are known to be influenced by various heritable factors with the effect ranges of 20% to 77%. In this study, we investigated a total of 10,716 Japanese individuals for the effects of genetic variations on regulation of the serum levels of the major protein components such as total protein (TP), albumin (ALB), and non-albumin protein (NAP) by a genome-wide association study (GWAS) and replication analysis. We identified one genetic locus significantly associated with both TP and NAP, one locus with ALB, and two loci with NAP. The variants associated with NAP are located within genetic loci encompassing genes that have important biological roles in the immune system, and also demonstrated significant associations with subsets of serum immunoglobulins using data of 1,600 additional Japanese individuals. These results should provide novel knowledge for the genetic background of the inter-individual variations of these serum protein levels.

Introduction

Serum proteins possess various biological functions such as hormones, enzymes, antibodies, and clotting agents, and some of them are valuable biomarkers that reflect several disease conditions. Major components of serum proteins are ALB (approximately 60%), globulins (mainly as γ–globulins, approximately 30%), and fibrinogens. Total serum protein level ranges from 6.5 to 8.5 g/dl and reveals significant inter-individual variations. The variations in serum levels are found to be influenced by various environmental factors. However, genetic factors are also known to affect its levels although the range of genetic effects varies by the reports from 20% to 77% [1]. Genome-wide association studies (GWAS) recently demonstrated that serum levels of several serum proteins can be strongly influenced by common genetic variants by *cis* or *trans* effects [2-4].

We previously reported the GWAS results of the hematological and biochemical traits in the Japanese population, including TP and ALB [5]. An associated SNP for TP, rs4273077 (P-value = 4.5×10^{-10}), is located in an intron of *TNFRSF13B* (Tumor Necrosis Factor Receptor Superfamily member 13B) that encodes TACI (transmembrane activator and calcium-modulator and cytophilin interactor), one of three TNF-receptor family members (BAFF-R, TACI, and BCMA) [6]. However, since rs4273077 showed no association with serum ALB levels (P = 0.089), we suspected that *TNFRSF13B* would have genetic effects primarily on the levels of the non-albumin fraction. TACI is expressed mainly in activated B cells and binds with a high affinity to two TNF ligands, APRIL (a Proliferation-Inducing Ligand, encoded by *TNFSF13*) and BAFF (B Cell-Activating Factor, encoded by *TNFSF13B*) [7]. TACI is implicated its involvement in B-cell homeostasis (including their survival, activation, and differentiation), immunoglobulin production, and antibody class switching [8-10]. Hence, the association of variants in *TNFRSF13B* with TP is likely to reflect the serum levels of immunoglobulins.

The aim of this study is to identify the genetic variations associated with serum levels of non-albumin proteins (NAP) levels, particularly those of immunoglobulins by GWAS of Japanese subjects.

Results

GWAS of total protein (TP), albumin (ALB), and non-albumin protein (NAP)

We conducted a GWAS using genotyping data and clinical information of 9,103 individuals who had been collected in the BioBank Japan Project as disease cohorts [11] (Table 1, Table S1). Genotyping was performed by the use of Illumina Human610-Quad BeadChip (Illumina, CA, USA). After applying stringent quality control (QC) filters for selection of individuals and SNPs (Materials and Methods), we additionally performed whole-genome imputation analysis using the data of HapMap Phase II East Asian populations and yielded the information of 2,178,644 autosomal SNPs with minor allele frequencies (MAF) of ≥ 0.01 and *Rsq* of ≥ 0.7. We then evaluated the association of the SNPs with the adjusted *Z* scores of serum levels of total protein (TP), albumin (ALB), and non-albumin protein (NAP). A Quantile-quantile (Q-Q) plot for each trait indicated low possibility of population stratification (inflation factors (λ_{GC}) for TP, ALB and NAP were 1.04, 1.02 and 1.02, respectively) (Figure S2).

Several highly-linked SNPs ($r^2 > 0.8$) in intronic regions of *TNFRSF13B* on chromosome 17p11.2 showed significant associations with both TP and NAP (rs4985726, $P = 2.8 \times 10^{-12}$ and 2.4×10^{-22}, respectively) (Table 2, Table S2, and Figure 1A and 1B). In addition, rs3803800 and rs11552708 in coding regions of *TNFSF13* on chromosome

17p13.1 demonstrated significant associations with NAP (P = 1.8 x 10^{-12} and 7.0 x 10^{-9}, respectively) (Table 2 and Figure 1B).

Since *TNFSF13* encodes APRIL, a ligand of TACI encoded by *TNFRSF13B*, the ligand-receptor interaction is likely to play a critical role in regulation of the serum NAP levels. However, we did not find any synergistic effects between SNPs in the receptor and ligand on NAP levels.

Rs10007186 located near *ANXA3* (annexin A3) on chromosome 4q21.2 also revealed the significant association with NAP (P = 3.3x10^{-9}), and a cluster of highly linked SNPs near the 5\` of *AFF3* (AF4/FMR2 family, member 3) on 2q11.2 indicated suggestive associations with NAP (rs4851274, P = 9.95x10^{-8}) (Table S2). For serum ALB, SNPs rs1260326 (in an exon) and rs3817588 (in an intron) in *GCKR* (glucokinase regulator) on 2p23.3 revealed significant associations (P = 3.4x10^{-8}, and 4.1x10^{-8}, respectively) (Table 2 and Table S2).

Conditional logistic regression analysis for the SNPs on 17p13.1 indicated that both rs3803800 and rs11552708 conferred independent associations with NAP levels when adjusted each other (P <0.023). These two SNPs were in linkage disequilibrium (LD; D\`= 0.99, r^2= 0.30), and the haplotype analysis of these two SNPs identified that a haplotype (rs3803800 [A] – rs11552708 [G]) revealed stronger association with NAP than single SNPs (P = 2.59x10^{-13}) (Table S3). Similarly, rs1260326 and rs3817588 in *GCKR* exhibited independent associations with ALB levels (P < 0.022), and were in LD (D\` = 0.95, r^2= 0.50). Besides, the haplotype (rs1260326 [C] – rs3817588 [C]) indicated stronger association with serum ALB (P = 2.83 x 10^{-9}) (Table S4). For 17p11.2 and 4q21.2 loci, no SNP remained to be significant after accounting for the effect of marker SNPs (rs4985726, and rs10007186, respectively).

When we examined genetic contribution of these variances for the traits, the combinations of these SNPs indicated above could explain nearly 0.5%, 2.3%, and 0.3% of variations of serum TP, NAP, and ALB, respectively.

Replication study

To validate the GWAS results, we performed a replication study using an independent set of ~1,600 subjects from the BioBank Japan [11] (Table 1). For each trait, we selected the marker SNP for the replication analysis at each locus that indicated the genome-wide significant level of 5.0×10^{-8} (rs4985726 in *TNFRSF13B*, rs3803800 in *TNFSF13*, rs1260326 in *GCKR*, and rs10007186 on 4q21.2). In addition, the two SNPs that remained to be significant after accounting for the effect of each marker SNP in two loci (rs11552708 in *TNFSF13* and rs3817588 in *GCKR*) were also further investigated.

SNPs rs4985726 in the *TNFRSF13B* locus as well as rs3803800 and rs11552708 in the *TNFSF13* locus revealed significant associations with both TP and NAP (Table 2). The association of rs1260326 in *GCKR* with serum ALB was also replicated ($P = 0.029$; Table 2). Meta-analyses combining the GWAS and the replication study yielded stronger associations of these SNPs than the GWAS (Table 2).

Rs10007186 near *ANXA3* revealed a suggestive association in the replication study ($P = 0.065$), and meta-analyses indicated that the association was unlikely to be false positive ($P = 1.3 \times 10^{-9}$).

Association of the SNPs identified in the GWAS of NAP with serum immunoglobulin isotypes

Immunoglobulin isotypes constitute the major components of NAP. Hence, we further examined the NAP-associated SNPs in the GWAS (*TNFRSF13B*, *TNFSF13*, and *ANXA3*) for the association with various serum immunoglobulins using the samples in the BioBank Japan [11] (IgG: n = 1,794, IgA: n = 1,675, IgM: n = 1,649, and IgE: n = 549; Table 1).

We found significant associations of rs4985726 in *TNFRSF13B* as well as rs3803800 and rs11552708 in *TNFSF13* with serum levels of IgG (P < 0.0023) and IgM (P < 0.018) (Table 3). For IgA, rs3803800 and rs11552708 in *TNFSF13* also revealed the significant association (P < 0.013), while rs4985726 in *TNFRSF13B* revealed no association (P = 0.099) (Table 3). Rs10007186 near *ANXA3* indicated significant association with IgA (P = 0.036), IgM (P = 0.019), and IgE ($P = 4.9 \times 10^{-4}$). However, these associated SNPs explained only 1.4%, 0.9%, 1.3%, and 2.0% of the variances of log-transformed values of serum IgG, IgA, IgM, and IgE, respectively.

Discussion

On the basis of the information of 10,716 Japanese individuals, we identified one genetic locus (*TNFRSF13B*) on chromosome 17p11.2 to be associated with both TP and NAP, two loci (*TNFSF13* on 17p13.1 and a region near *ANXA3* on 4q21.2) with NAP, and one locus (*GCKR*) on 2p23.3 with ALB with the genome-wide significant level.

The marker SNP rs4985726 showing the association with TP and NAP is located in an intron of *TNFRSF13B* on chromosome 17p11.2. A possible mechanism of the association between this SNP and those traits could be explained by its strong LD with rs34562254 (D` = 1, r^2 = 0.97) that exhibits a missense variation (C>T, Pro251Leu) located

in the intracellular domain of the receptor molecule. The *in silico* prediction of the amino acid substitution by rs34562254 in the PolyPhen-2 and SNPinfo database [12,13] suggested a “probably damaging” effect on the protein structure.

SNPs in *TNFSF13* (encoding APRIL) that were identified their association with NAP are missense variants, rs3803800 (A>G, Asn96Ser) and rs11552708 (G>A, Gly67Arg). APRIL was first described to have a promoter function for tumor-cell proliferation and survival [14]. APRIL is cleaved in the Golgi apparatus by furin at a 104Arg/105Ala site [15], and interestingly, rs3803800 is closely located to this cleavage site. Hence, this SNP might affect the cleavage affinity. The other possibility is the effect on splicing because both SNPs are predicted to be located within binding sites of splicing regulatory elements. However, further depth investigation should be required to elucidate these possibilities

The SNP rs4985726 in *TNFRSF13B* as well as rs3803800 and rs11552708 in *TNFSF13* also revealed significant associations with serum levels of IgG, IgA, and IgM. It is notable that the two genes encode a TNF-receptor and ligand axis that serves important roles for mediation of the antibody class switching and for regulation of immunoglobulin production [8,9]. Furthermore, knockout mice of either *TNFRSF13B* or *TNFSF13* presented a common phenotype of IgA deficiency with the impaired antibody response to T cell-independent antigens [16]. In addition, mutations in *TNFRSF13B* were reported in cases with common variable immunodeficiency (CVID; MIM # 607594) and selective IgA deficiency (IGAD; MIM # 137100) [17]. A combination of these significant statistical and biological evidences would suggest that the association of these two loci with NAP was reflecting at least their associations with regulation of serum immunoglobulin levels. It is also known that immunoglobulins are the major components of the NAP, which provides a compelling evidence for our results. The facts that both SNPs rs3803800 [A] and rs11552708 [G] in *TNFSF13* reported their associations with the susceptibility to the

Systemic Lupus Erythematosus (SLE) in the Japanese population and that high serum APRIL was detected in the sera of individuals with the rs3803800 [A]–rs11552708 [G] haplotype [18] further support the significance of these SNPs in the regulation of immunoglobulin. In this study, we observed that possession of two copies of SLE-risk alleles revealed higher serum levels of NAP, IgG, IgA, and IgM (Figure S3), which provide a good example of genetic loci which influence both susceptibility to complex diseases and quantitative traits.

Rs10007186 associated with NAP (P_{meta} = 1.3 x 10^{-9}) is located about 57.4 kb downstream to *ANXA3* encoding annexin A3, a member of annexin family of calcium-dependent phospholipid-binding proteins [19]. Annexin A3 was found to be translocated into phagosomes in dendritic cells [20], which are antigen-presenting cells serve as messengers between innate and adaptive immune response, and play a key role in allergic, inflammatory, and autoimmune conditions. In addition, annexin A3 was also found to be associated with neutrophils granule membranes [21], where it can play a regulatory role in calcium-dependent granule secretions that contribute to acute inflammation and chronic tissue destruction. The association of rs10007186 with IgA, IgM, and IgE, would suggest additional biological roles of annexin A3 in the immune response.

We also confirmed the association of SNPs in *GCKR* with serum ALB level by the GWAS and the replication study (rs1260326, P_{meta} = 3.1 x 10^{-9}). Rs1260326 is a missense variant (T>C, Leu446Pro) and predicted to cause a damaging effect on the protein structure. *GCKR* is a common locus associated with several metabolic traits [4,22-24] and rs1260326 has been reported its association with serum triglycerides [4].

As a conclusion, the present study identified genetic loci that influence the inter-individual variations of serum levels of TP, ALB, and NAP. The associated loci with NAP

encompassing genes which are implicated their biological roles in the immune system including the TNF-receptor and its ligand, and their associations with immunoglobulin isotypes were demonstrated. Our results should add novel insight to understand the genetic background contributing to the regulation of the serum levels of NAP and its major components.

Materials and Methods

Study cohorts

For the GWAS, 9,103 subjects derived from 10 disease cohorts (colorectal cancer, breast cancer, prostate cancer, lung cancer, gastric cancer, diabetes mellitus, peripheral artery disease, atrial fibrillation, ischemic stroke, and myocardial infarction) were selected , and for the replication study, we used data from >1,600 independent individuals selected from more than 20 other cohorts from the BioBank Japan [11] (Table 1 and Table S1). For immunoglobulin isotypes analyses, the data from ~1,600 additional individuals in the BioBank Japan [11] was used (Table 1). The clinical information for the samples is updated annually using a standard questionnaire in the 66 hospitals participating in the project. Written informed consent was obtained from all subjects. The research project was approved by the ethical committees in the Institute of Medical Science, the University of Tokyo, and the Center of Genomic Medicine, RIKEN, Yokohama, Japan.

Genotyping and quality control (Q.C) filters

In the GWAS, SNPs were genotyped using Illumina HumanHap610-Quad BeadChip (Illumina, CA, USA). After the exclusion of the samples with call rates of <0.98, we excluded closely related individuals (in 1st or 2nd degree kinships) using identity-by-descent (IBD)

evaluated by PLINK version 1.0.6 [25]. We also excluded individuals who were outliers in the cluster analysis using principle component analysis performed by EIGENSTRAT 3.0 along with HapMap Phase II populations (Figure S1). In addition, SNPs with call rates of <0.99, MAF of < 0.01 and Hardy Weinberg equilibrium of P <1.0 x 10^{-7} were excluded.

Genotyping data of the SNPs selected for replication analyses and for testing with immunoglobulin levels were generated by using multiplex PCR-based Invader Assay (Third Wave Technologies, Madison, WI, USA) [26]. Genotypes were judged by visual inspection, following the application of QC measures of individuals call rates of >98% and call rates of >99% of individuals. We could not obtain the genotype data of rs3817588 in *GCKR* using the Invader assay.

Whole-genome imputation of genotypes

We performed whole-genome imputation of the GWAS subjects in a two-step procedure, as described elsewhere [27]. HapMap phase II Japanese (JPT) and Han Chinese (HCB) individuals (release 24) were adopted as reference panels. We excluded the imputed SNPs with MAF of <0.01 or *Rsq* of <0.7. As a result, a total of 2,178,644 autosomal SNPs were used for the GWAS.

Statistical analysis

We obtained the non-transformed values of TP, ALB and NAP (mg/dl) of the subjects from the clinical information stored in the BioBank Japan [11], and adjusted them in linear regression models with age, gender, body mass index (BMI), smoking, drinking status, and

affection status of the disease as covariates. The residuals were then normalized as *Z* scores and subjects with *Z* scores of <-4 or >4 were removed from each trait analysis. The associations of the SNPs with *Z* scores were evaluated in linear regression models assuming additive effects of the allele dosages, using mach2qtl software. The same methods of the data normalization and statistical models were applied for the replication analyses and for testing the association with common log-transformed values of immunoglobulin isotypes (IgG IgA, IgM, and IgE). Meta-analyses of the GWAS and the replication study were performed using the inverse-variance method assuming fixed-effects model.

The haplotype analyses were performed using the Haplo Stats package (version 1.4.0) implemented in *R* statistical software. Epistatic effects of the SNPs in *TNFRSF13B* and *TNFSF13* were evaluated by using linear regression model incorporating the product of the allele dosages of the SNPs in the loci as an independent variable. All statistical analyses including haplotype analyses were performed using the *R* statistical software version 2.9.1 except for genome-wide linear regression analyses. LD analyses were performed using Haploview 4.2 software, PLINK, and SNAP database.

Web resources:

The URLs for the data presented in this paper are as follows:

The BioBank Japan Project, http://biobankjp.org/

PLIKN software, http://pngu.mgh.harvard.edu/~purcell/plink/

EIGENSTRAT software, http://genepath.med.harvard.edu/~reich/EIGENSTRAT.htm

The International HapMap Project, http://www.hapmap.org/

MACH and mach2qtl software, http://www.sph.umich.edu/csg/abecasis/MaCH/index.html

R statistical environment, http://www.r-project.org/

Haploview software, www.broad.mit.edu/mpg/haploview/

SNAP, http://www.broadinstitute.org/mpg/snap/ldsearch.php

Locus Zoom, http://csg.sph.umich.edu/locuszoom/

Acknowledgments

We thank all subjects participating in this study for providing their clinical data and DNA samples. We acknowledge the staff in the BioBank Japan [11] and the members of the Laboratory of the Genotyping Development at RIKEN Center for Genomic Medicine for their technical support.

Author contributions

Conceived the study: Yusuke Nakamura, Koichi Matsuda, Yukinori Okada; designed the experiments: Yusuke Nakamura, Koichi Matsuda, Yukinori Okada, Yoichiro Kamatani, Michiaki Kubo, Wael Osman; performed the experiments: Wael Osman, Michiaki Kubo; analyzed the data: Yukinori Okada, Yoichiro Kamatani, Wael Osman; contributed reagents/materials/analysis tools: Yusuke Nakamura, Koichi Matsuda, Yukinori Okada, Michiaki Kubo; summarized the whole results: Wael Osman; wrote the manuscript: Wael Osman, Yukinori Okada, Yusuke Nakamura.

Conflict of interest statement

The authors declare no conflict of interest. This work was supported by Leading Project for Personalized Medicine in the Ministry of Education, Culture, Sports, Science and Technology, Japan. The funders had no role in study design, data collection and analysis, decision to publish, or preparation of the manuscript.

References

1. Rahmioglu N, Andrew T, Cherkas L, Surdulescu G, Swaminathan R, et al. (2009) Epidemiology and genetic epidemiology of the liver function test proteins. PLoS One 4: e4435.
2. Melzer D, Perry J, Hernandez D, Corsi A, Stevens K, et al. (2008) A genome-wide association study identifies protein quantitative trait loci (pQTLs). PLoS Genet 4: e1000072.
3. Weidinger S, Gieger C, Rodriguez E, Baurecht H, Mempel M, et al. (2008) Genome-wide scan on total serum IgE levels identifies FCER1A as novel susceptibility locus. PLoS Genet 4: e1000166.
4. Sabatti C, Service SK, Hartikainen AL, Pouta A, Ripatti S, et al. (2009) Genome-wide association analysis of metabolic traits in a birth cohort from a founder population. Nat Genet 41: 35-46.
5. Kamatani Y, Matsuda K, Okada Y, Kubo M, Hosono N, et al. (2010) Genome-wide association study of hematological and biochemical traits in a Japanese population. Nat Genet 42: 210-215.
6. Bossen C, Schneider P (2006) BAFF, APRIL and their receptors: structure, function and signaling. Semin Immunol 18: 263-275.
7. Mackay F, Kalled S (2002) TNF ligands and receptors in autoimmunity: an update. Curr Opin Immunol 14: 783-790.
8. Castigli E, Wilson S, Scott S, Dedeoglu F, Xu S, et al. (2005) TACI and BAFF-R mediate isotype switching in B cells. J Exp Med 201: 35-39.
9. Sakurai D, Hase H, Kanno Y, Kojima H, Okumura K, et al. (2007) TACI regulates IgA production by APRIL in collaboration with HSPG. Blood 109: 2961-2967.

10. Mackay F, Schneider P (2008) TACI, an enigmatic BAFF/APRIL receptor, with new unappreciated biochemical and biological properties. Cytokine Growth Factor Rev 19: 263-276.

11. Nakamura Y (2007) The BioBank Japan Project. Clin Adv Hematol Oncol 5: 696-697.

12. Adzhubei IA, Schmidt S, Peshkin L, Ramensky VE, Gerasimova A, et al. (2010) A method and server for predicting damaging missense mutations. Nat Methods 7: 248-249.

13. Xu Z, Taylor JA (2009) SNPinfo: integrating GWAS and candidate gene information into functional SNP selection for genetic association studies. Nucleic Acids Res 37: W600-605.

14. Hahne M, Kataoka T, Schröter M, Hofmann K, Irmler M, et al. (1998) APRIL, a new ligand of the tumor necrosis factor family, stimulates tumor cell growth. J Exp Med 188: 1185-1190.

15. López-Fraga M, Fernández R, Albar JP, Hahne M (2001) Biologically active APRIL is secreted following intracellular processing in the Golgi apparatus by furin convertase. EMBO Rep 2: 945-951.

16. Castigli E, Scott S, Dedeoglu F, Bryce P, Jabara H, et al. (2004) Impaired IgA class switching in APRIL-deficient mice. Proc Natl Acad Sci U S A 101: 3903-3908.

17. Castigli E, Wilson S, Garibyan L, Rachid R, Bonilla F, et al. (2005) TACI is mutant in common variable immunodeficiency and IgA deficiency. Nat Genet 37: 829-834.

18. Koyama T, Tsukamoto H, Masumoto K, Himeji D, Hayashi K, et al. (2003) A novel polymorphism of the human APRIL gene is associated with systemic lupus erythematosus. Rheumatology (Oxford) 42: 980-985.

19. Gerke V, Moss SE (2002) Annexins: from structure to function. Physiol Rev 82: 331-371.

20. Larsson M, Majeed M, Ernst JD, Magnusson KE, Stendahl O, et al. (1997) Role of annexins in endocytosis of antigens in immature human dendritic cells. Immunology 92: 501-511.

21. Rosales JL, Ernst JD (1997) Calcium-dependent neutrophil secretion: characterization and regulation by annexins. J Immunol 159: 6195-6202.

22. Ridker PM, Pare G, Parker A, Zee RY, Danik JS, et al. (2008) Loci related to metabolic-syndrome pathways including LEPR,HNF1A, IL6R, and GCKR associate with plasma C-reactive protein: the Women's Genome Health Study. Am J Hum Genet 82: 1185-1192.

23. Kolz M, Johnson T, Sanna S, Teumer A, Vitart V, et al. (2009) Meta-analysis of 28,141 individuals identifies common variants within five new loci that influence uric acid concentrations. PLoS Genet 5: e1000504.

24. Orho-Melander M, Melander O, Guiducci C, Perez-Martinez P, Corella D, et al. (2008) Common missense variant in the glucokinase regulatory protein gene is associated with increased plasma triglyceride and C-reactive protein but lower fasting glucose concentrations. Diabetes 57: 3112-3121.

25. Purcell S, Neale B, Todd-Brown K, Thomas L, Ferreira M, et al. (2007) PLINK: a tool set for whole-genome association and population-based linkage analyses. Am J Hum Genet 81: 559-575.

26. Olivier M (2005) The Invader assay for SNP genotyping. Mutat Res 573: 103-110.

27. Okada Y, Hirota T, Kamatani Y, Takahashi A, Ohmiya H, et al. (2011) Identification of nine novel loci associated with white blood cell subtypes in a Japanese population. PLoS Genet 7: e1002067.

Figure legends:

Figure 1. **Manhattan plots for the GWAS of (A) TP, (B) NAP and (C) ALB.** SNPs were plotted based on their physical chromosomal positions (horizontal axis) together with their –$\log_{10}$ (*P*-values) in the GWAS (vertical axis). The black horizontal line shows the genome-wide significance threshold of P = 5.0 x 10^{-8}. The SNPs for which *P*-values were smaller than 1.0 x 10^{-15} are indicated at the upper limit of the plots.

Figure 2. Regional plots for the associations of the SNPs in the GWAS stage of TP, ALB and NAP. SNPs plotted with their –$\log_{10}$ (*P*-values) in the GWAS based on their physical chromosomal positions. Genotyped SNPs are indicated as circles, while imputed SNPs are indicated as triangles. The color scheme indicated the linkage disequilibrium displayed as r^2 values between all SNPs and the top-ranked SNP in each plot. The tested trait, chromosomal locus, and the top-ranked SNPs (in purple color) in the GWAS and combined analyses together with their *P*-values are shown in each plot. The blue lines represent the recombination rates estimated based on HapMap Phase II database. The plots were drawn using Locus Zoom software.

Supporting information:

Figure S1.

Principal component analysis Plot of cohorts included in the GWAS. All individuals who were finally incorporated in the GWAS together with the four populations in the HapMap Phase II database (Japanese: JPT; Han Chinese: HCB; Africans: YRI, and European: CEU) were plotted based on the first two eigenvectors.

Figure S2.

Quantile-Quantile (Q-Q) plots for the GWAS of (A) TP, (B) NAP and (C) ALB. The inflation factor, λ_{GC}, for the analysis is shown in the legend of each plot. The SNPs for which *P*-values were smaller than 1.0×10^{-15} are indicated at the upper limit of the plots.

Figure S3.

Relationship between the genotypes of SNPs identified in the study and the levels of tested proteins: (A) rs4985726, (B) rs3803800, (C) rs11552708, (D) rs10007186, and (E) rs1260326. For each box plot, the bold line indicates the median value which is the 50th quartile. The limits of each box are the 25th and 75th quartiles.

Table S1. Characteristics of the GWAS cohorts.

Table S2. SNPs showed suggestive associations with each trait ($P < 1.0 \times 10^{-6}$).

Table S3. Haplotype analysis of rs3803800 and rs11552708 in *TNFSF13* in association with NAP.

Table S4. Haplotype analysis of rs1260326 and rs3817588 in *GCKR* in association with ALB.

Table 1. Characteristics of the proteins analyzed in this study

	TP		ALB		NAP					
	GWAS	Replication	GWAS	Replication	GWAS	Replication	IgG *	IgA *	IgM *	IgE *
No.	9,090	1,626	9,103	1,607	9,077	1,629	1,794	1,675	1,649	549
M ± S.D [a]	7.10 ± 0.50	7.06 ± 0.73	4.25 ± 0.35	4.00 ± 0.51	2.85 ± 0.42	3.07 ± 0.57	1.44 ± 0.61	0.27 ± 0.15	0.11 ± 0.07	1306.54 ± 5598.06
Age [b]	69.52 ± 10.44	59.52 ± 15.43	69.52 ± 10.44	59.54 ± 15.39	69.51 ± 10.44	59.48 ± 15.52	59.70 ± 15.46	59.38 ± 15.73	59.42 ± 15.57	62.54 ± 18.61
Female %	37.45	45.08	37.41	45.12	37.46	45.12	55.30	54.57	54.88	63.93
BMI [b]	22.91 ± 3.45	23.31 ± 5.67	22.91 ± 3.45	23.34 ± 5.69	22.91 ± 3.45	23.29 ± 5.67	23.17 ± 5.00	23.20 ± 5.09	23.19 ± 5.07	22.73 ± 4.22
Smokers %	42.11	51.91	42.11	52.15	42.05	51.81	51.90	51.82	52.27	48.63
Drinkers %	29.37	51.97	29.37	52.08	29.40	51.81	51.00	50.81	50.82	41.35

[a] M ± S.D: mean value ± standard deviation of each protein is indicated in g/dl except for IgE, which is indicated as IU/ml.

[b] Age and body mass index (BMI) are shown as mean values ± standard deviation.

* Log-transformed values were applied in the analysis.

Abbreviations: GWAS: genome-wide association study, TP: total protein, ALB: albumin, NAP: non-albumin protein.

Table 2. Summary results of the GWAS and the replication study of TP, ALB, and NAP.

		Chr:	Nearest	A1/		GWAS		Replication		Meta analysis		% variance
Trait	SNP	Position	gene	A2[a]	MAF	Effect [b] (s.e)	*P* [c]	Effect [b] (s.e)	*P* [c]	Effect [b] (s.e)	*P* [c]	explained
**TP**	rs4985726*	17:16804363	*TNFRSF13B*	C/G	0.375	0.108 (0.015)	2.8×10^{-12}	0.100 (0.030)	0.0010	0.107 (0.0138)	1.2×10^{-14}	0.53
**ALB**	rs1260326	2: 27584444	*GCKR*	T/C	0.445	-0.082 (0.015)	3.4×10^{-8}	-0.070 (0.032)	0.029	-0.080 (0.014)	3.1×10^{-9}	0.32
**NAP**	rs4985726*	17:16804363	*TNFRSF13B*	C/G	0.375	0.148 (0.015)	2.4×10^{-22}	0.090 (0.028)	0.0013	0.135 (0.013)	7.1×10^{-24}	1.03
	rs3803800	17:7403693	*TNFSF13*	G/A	0.311	0.108 (0.015)	1.8×10^{-12}	0.090 (0.029)	0.0022	0.104 (0.013)	7.2×10^{-15}	0.53
	rs11552708	17:7403279	*TNFSF13*	G/A	0.401	-0.084 (0.015)	7.0×10^{-9}	-0.070 (0.027)	0.0091	-0.081 (0.013)	7.5×10^{-10}	0.36
	rs10007186*	4:79808069	*ANXA3*	T/C	0.307	0.095 (0.016)	3.3×10^{-9}	0.053 (0.029)	0.065	0.085 (0.014)	1.3×10^{-9}	0.38

[a] A1/A2: major/minor alleles.

[b] The effect of the minor allele on the normalized values based on an additive genetic model.

[c] For the GWAS and replication analysis, *P*-values were obtained by linear regression test model, for the Meta analysis by inverse-variance method.

*SNPs obtained by whole-imputation analysis.

Abbreviations: GWAS: genome-wide association study, MAF: minor allele frequency, TP: total protein, ALB: albumin, NAP: non-albumin protein, s.e: standard error.

Table 3. Association of the SNPs in the GWAS of the NAP with immunoglobulin isotypes

		IgG			IgA			IgM			IgE		
SNP	Gene	Effect [a] (s.e)	*P* [b]	%EV	Effect [a] (s.e)	*P* [b]	%EV	Effect [a] (s.e)	*P* [b]	%EV	Effect [a] (s.e)	*P* [b]	%EV
rs4985726	*TNFRSF13B*	0.071 (0.022)	1.4×10^{-3}	0.51	0.049 (0.030)	0.099	–	-0.090 (0.032)	5.9×10^{-3}	0.40	0.039 (0.064)	0.54	–
rs3803800	*TNFSF13*	-0.074 (0.024)	2.2×10^{-3}	0.47	-0.086 (0.031)	6.2×10^{-3}	0.39	-0.082 (0.034)	0.018	0.29	-0.117 (0.067)	0.080	–
rs11552708	*TNFSF13*	0.067 (0.022)	2.3×10^{-3}	0.46	0.072 (0.029)	0.013	0.31	0.078 (0.032)	0.014	0.31	0.059 (0.060)	0.33	–
rs10007186	*ANXA3*	-0.018 (0.022)	0.42	–	-0.063 (0.030)	0.036	0.20	-0.078 (0.033)	0.019	0.27	0.200 (0.057)	4.9×10^{-4}	2.02

[a] The effect of the minor alleles on the standardized values.

[b] *P*-values for the associations of SNPs with each normalized immunoglobulin isotype obtained by using a linear regression model.

Abbreviations: s.e: standard error, %EV: percentage of the explanatory variance.

Figure
Click here to download high resolution image

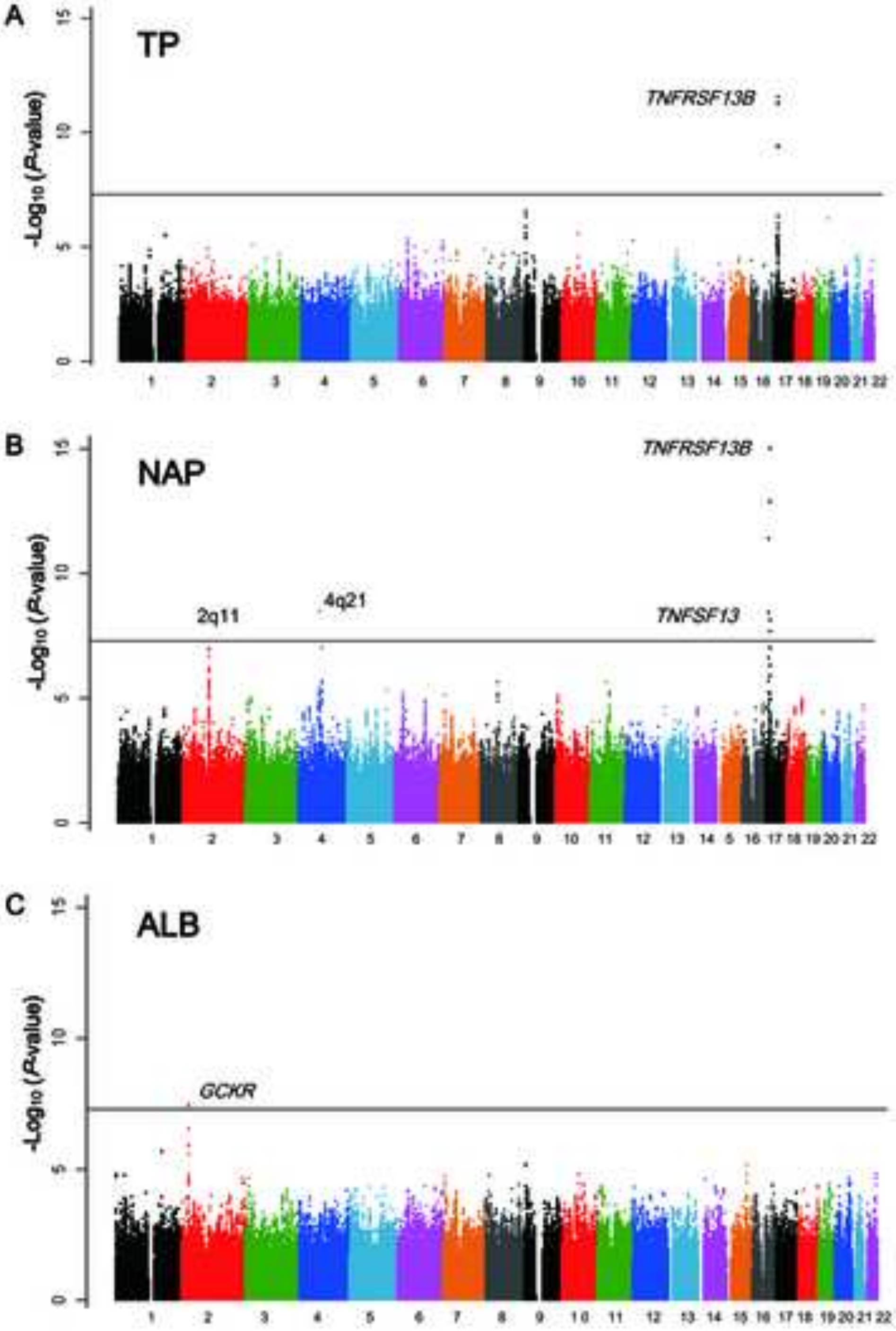

Figure
Click here to download high resolution image

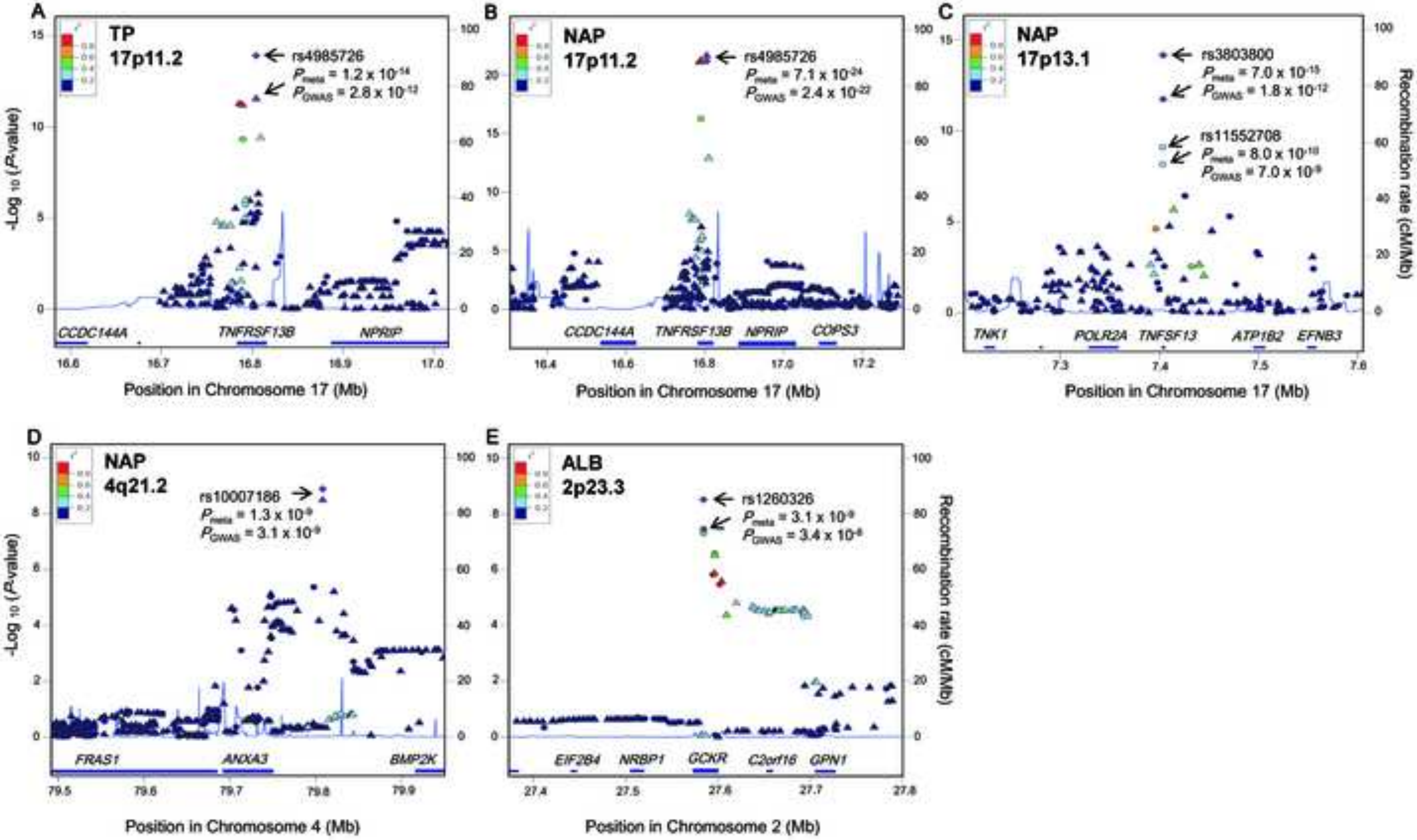

Supporting Information
Click here to download Supporting Information: Figure S1-Osman et al.pdf

Supporting Information
Click here to download Supporting Information: Figure S2-Osman et al.pdf

Supporting Information
Click here to download Supporting Information: Figure S3-Osman et al.pdf

Supporting Information
Click here to download Supporting Information: Table S1-Osman et al.doc

Supporting Information
Click here to download Supporting Information: Table S2-Osman et al.doc

Supporting Information

Click here to download Supporting Information: Table S3-Osman et al.doc

Supporting Information
Click here to download Supporting Information: Table S4-Osman et al.doc
