# Supplementary material for: Association of Common Variants in TNFRSF13B, TNFSF13, and ANXA3 with Serum Levels of Non-Albumin Protein and Immunoglobulin Isotypes in Japanese
Source: PLoS One. 2012 Apr 27;7(4):e32683. doi: 10.1371/journal.pone.0032683 (PMC3338726; doi:10.1371/journal.pone.0032683)
Supplement: Figure S3 — Relationship between the genotypes of SNPs identified in the study and the levels of tested proteins: (A) rs4985726, (B) rs3803800, (C) rs11552708, (D) rs10007186, and (E) rs1260326. For each box plot, the bold line indicates the median value which is the 50th quartile. The limits of each box are the 25th and 75th quartiles. (PDF) [file pone.0032683.s003.pdf]

(A) rs4985726; 0: CC, 1: CG, 2: GG

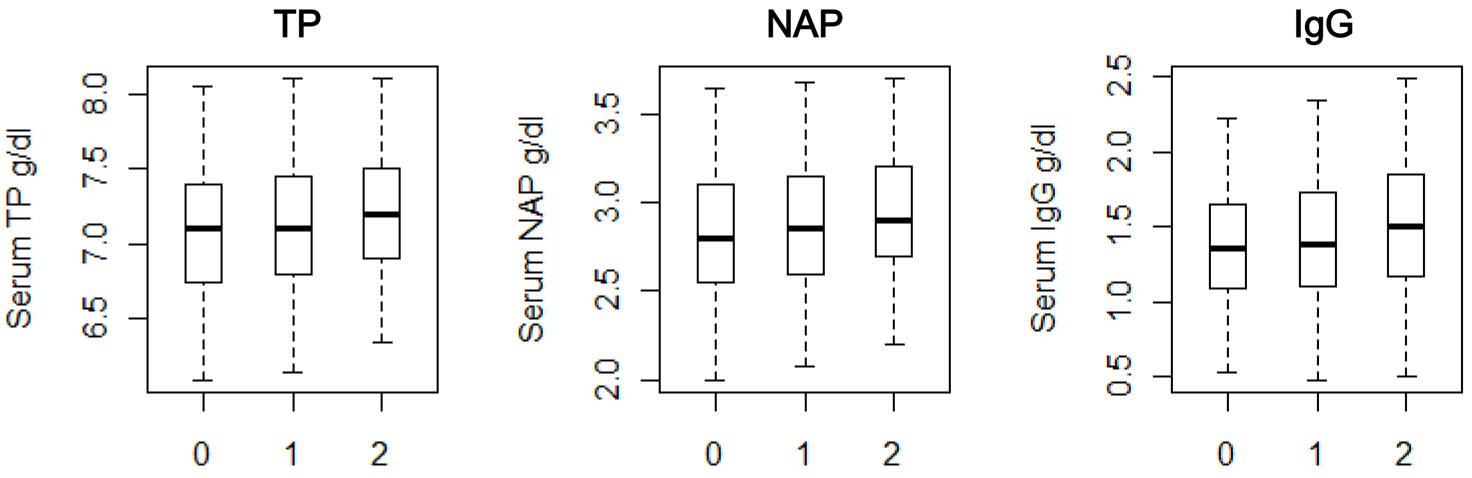

	TP 0	TP 1	TP 2	NAP 0	NAP 1	NAP 2	IgG 0	IgG 1	IgG 2
Number	4,167	4,962	1,563	4,166	4,964	1,565	644	874	244
Average/genotype (g/dl)	7.05	7.11	7.16	2.83	2.90	2.95	1.38	1.43	1.51

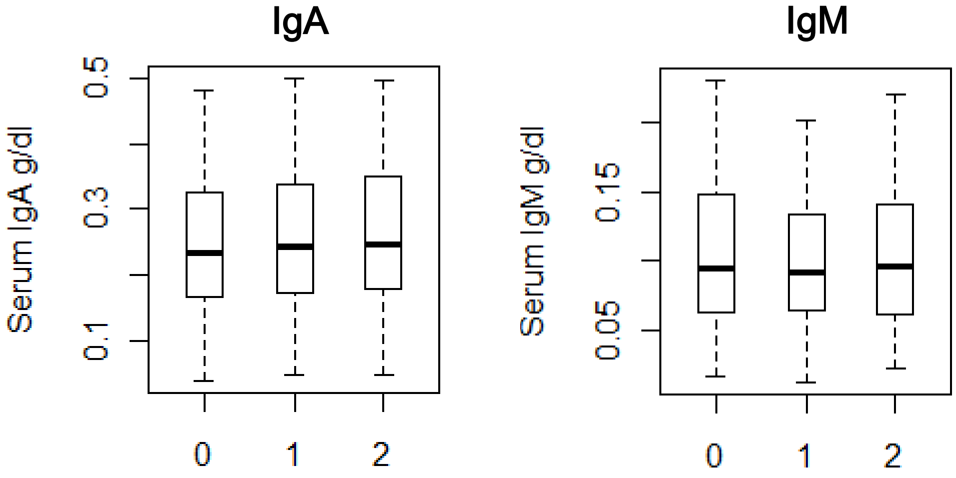

	IgA 0	IgA 1	IgA 2	IgM 0	IgM 1	IgM 2
Number	616	821	237	644	775	229
Average/genotype (g/dl)	0.26	0.28	0.27	0.12	0.11	0.11

(B) rs3803800; 0: AA, 1: AG, 2: GG

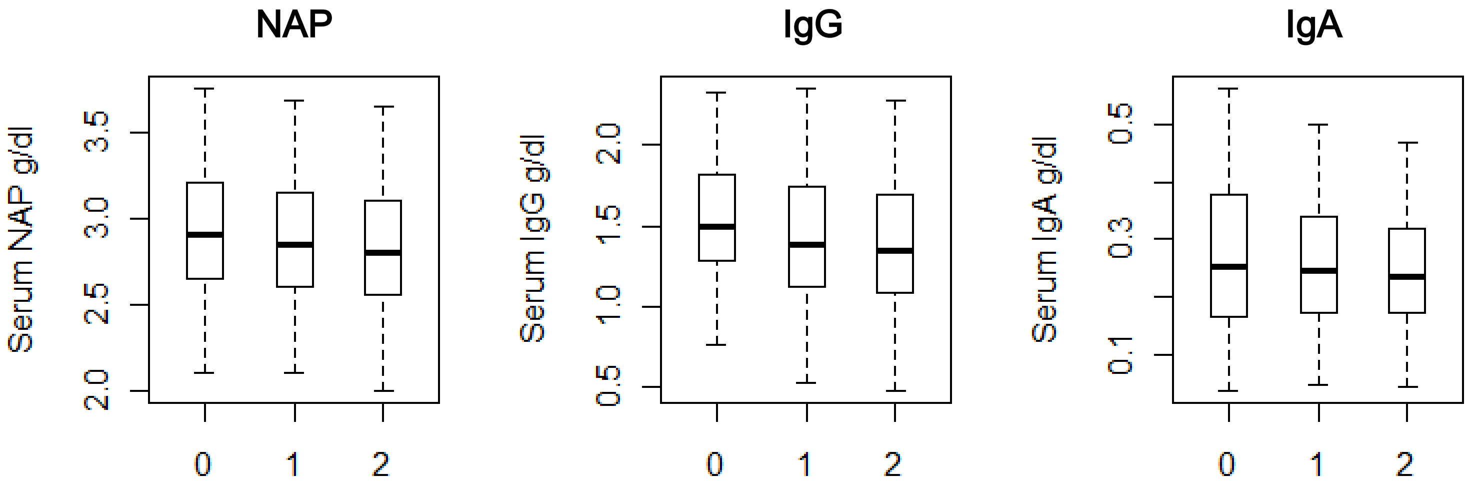

	NAP 0	NAP 1	NAP 2	IgG 0	IgG 1	IgG 2	IgA 0	IgA 1	IgA 2
Number	1,061	4,549	5,120	154	789	848	148	740	784
Average/genotype (g/dl)	2.94	2.90	2.85	1.63	1.44	1.41	0.29	0.28	0.26

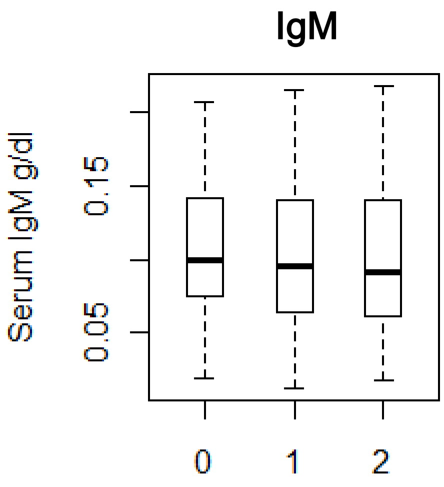

	0	1	2
Number	151	722	776
Average/genotype (g/dl)	0.12	0.11	0.11

(C) rs11552708; 0: AA, 1: AG, 2: GG

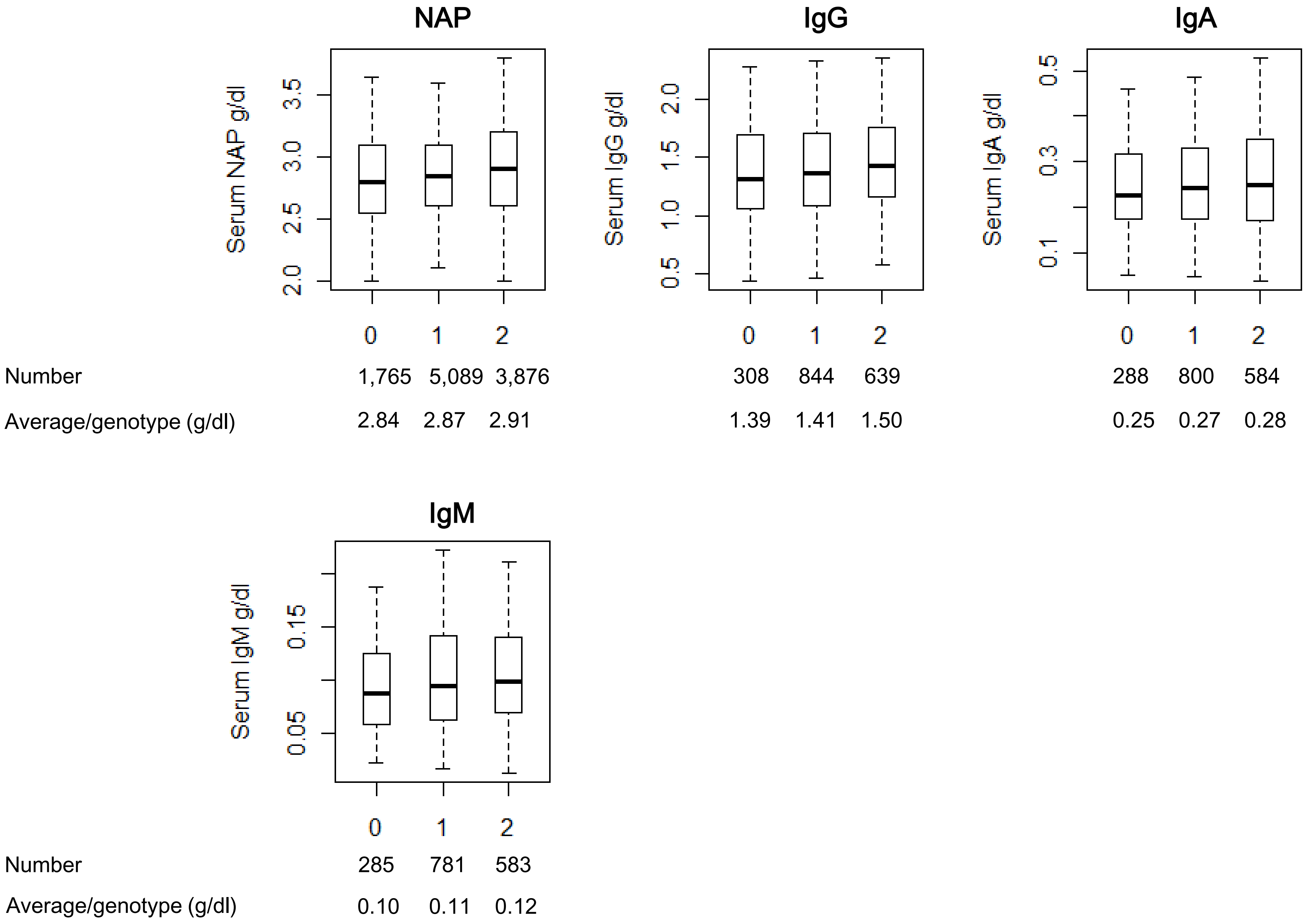

(D) rs10007186; 0: CC, 1: TC, 2: TT

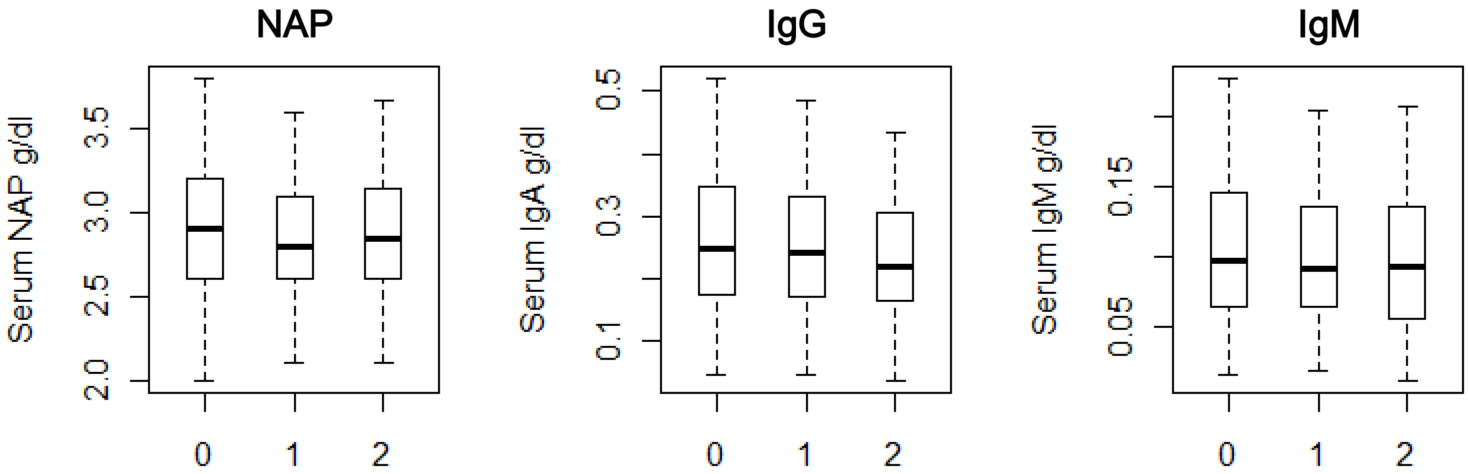

	0	1	2	0	1	2	0	1	2
Number	1,639	4,536	4,520	756	729	188	754	711	183
Average/genotype (g/dl)	2.94	2.86	2.88	0.27	0.27	0.25	0.12	0.11	0.10

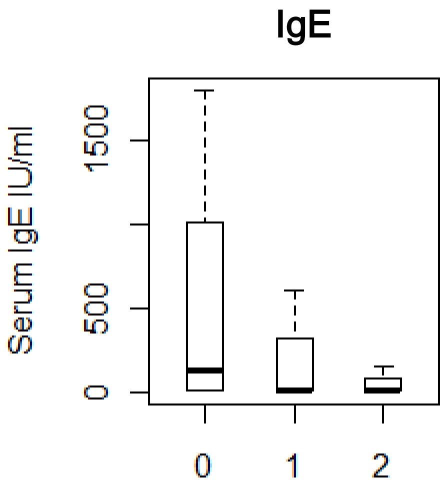

	0	1	2
Number	144	242	162
Average/genotype (IU/ml)	1646.82	1516.59	698.35

(E) rs1260326; 0: CC, 1: TC, 2: TT

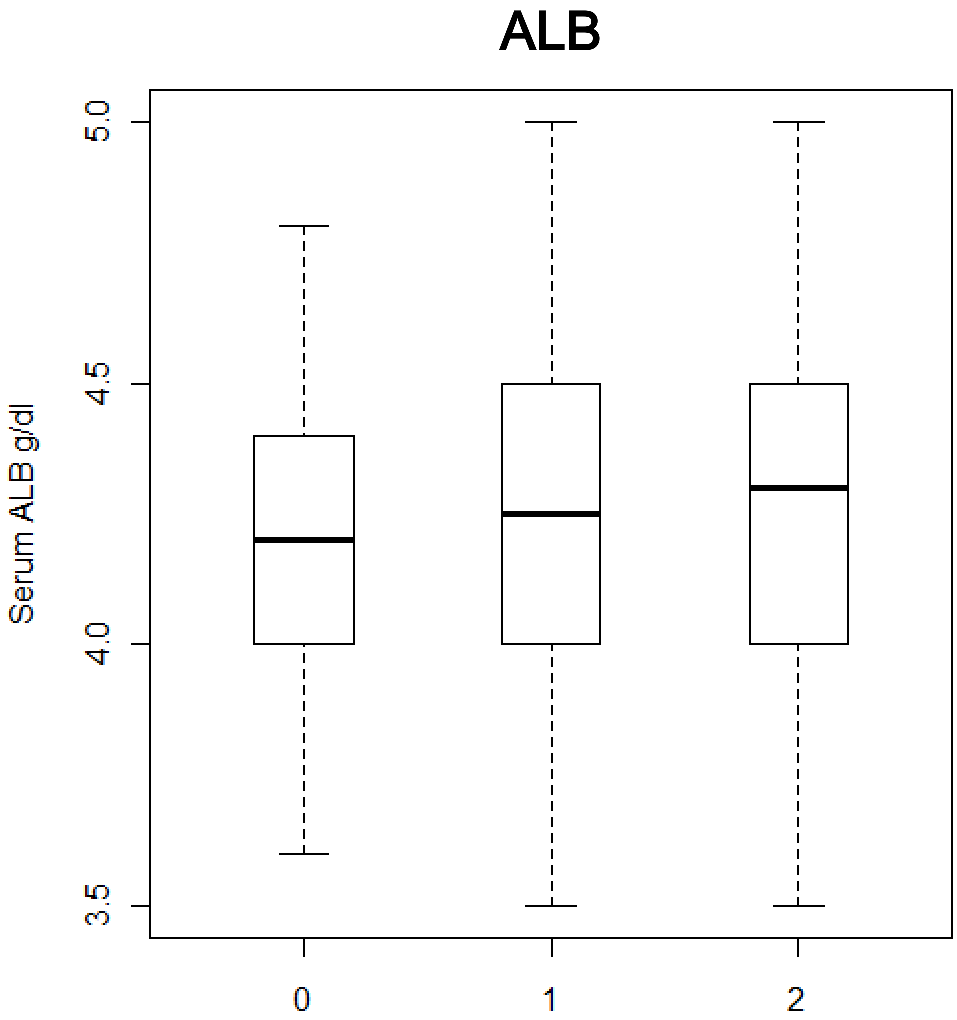

	0	1	2
Number	2,254	5,289	3,130
Average/genotype (g/dl)	4.18	4.21	4.26
