# Supplementary material for: Association of Common Variants in TNFRSF13B, TNFSF13, and ANXA3 with Serum Levels of Non-Albumin Protein and Immunoglobulin Isotypes in Japanese
Source: PLoS One. 2012 Apr 27;7(4):e32683. doi: 10.1371/journal.pone.0032683 (PMC3338726; doi:10.1371/journal.pone.0032683)
Supplement: Table S1 — Characteristics of the GWAS cohorts. (DOC) [file pone.0032683.s004.doc]

| | **Table S1. Characteristics of GWAS cohorts.** | | | | | | | | |  | | --- | --- | --- | --- | --- | --- | --- | --- | --- | --- | | Disease | No. | Age ^a^ | Female % | BMI ^a^ | Smokers (%) | | Drinkers (%) | | | | Colorectal cancer | 1,349 | 60.9 ± 11.0 | 40.2 | 22.5 ± 3.4 | 42.7 | | 32.3 | | | | Breast cancer | 1,346 | 54.8 ± 9.10 | 100 | 22.8 ± 3.6 | 17.0 | | 14.7 | | | | Prostate cancer | 1,096 | 69.0 ± 7.40 | 0.00 | 23.5 ± 2.8 | 39.2 | | 42.7 | | | | Lung cancer | 1,183 | 65.9 ± 9.30 | 36.6 | 22.3 ± 3.3 | 48.1 | | 31.8 | | | | Gastric cancer | 1,307 | 65.1 ± 8.60 | 19.7 | 21.3 ± 3.1 | 54.3 | | 36.0 | | | | Diabetes Mellitus | 758 | 65.3 ± 10.4 | 38.8 | 24.1 ± 3.7 | 40.6 | | 23.4 | | | | Peripheral Artery Disease | 197 | 70.7 ± 9.20 | 12.7 | 22.8 ± 3.2 | 72.1 | | 28.9 | | | | Atrial Fibrillation | 250 | 67.9 ± 10.1 | 27.6 | 24.1 ± 3.6 | 44.8 | | 32.8 | | | | Ischemic stroke | 997 | 67.6 ± 8.50 | 34.1 | 23.8 ± 3.3 | 39.7 | | 25.1 | | | | Myocardial infarction | 574 | 59.6 ± 10.4 | 12.7 | 24.4 ± 3.3 | 59.6 | | 26.3 | | | | ^a^ Age and body mass index (BMI) are shown as mean ± standard deviation. | | | |  | |  | |  |  | | All cohorts were genotyped using Illumina Human610-Quad BeadChip. | | | |  | |  | |  |  | |
| --- | --- | --- | --- | --- | --- | --- | --- | --- | --- | --- | --- | --- | --- | --- | --- | --- | --- | --- | --- | --- | --- | --- | --- | --- | --- | --- | --- | --- | --- | --- | --- | --- | --- | --- | --- | --- | --- | --- | --- | --- | --- | --- | --- | --- | --- | --- | --- | --- | --- | --- | --- | --- | --- | --- | --- | --- | --- | --- | --- | --- | --- | --- | --- | --- | --- | --- | --- | --- | --- | --- | --- | --- | --- | --- | --- | --- | --- | --- | --- | --- | --- | --- | --- | --- | --- | --- | --- | --- | --- | --- | --- | --- | --- | --- | --- | --- | --- | --- | --- | --- | --- | --- | --- | --- | --- | --- | --- | --- | --- | --- | --- | --- | --- | --- | --- | --- | --- | --- | --- | --- | --- | --- | --- | --- | --- | --- | --- | --- | --- | --- | --- | --- | --- | --- | --- | --- | --- | --- | --- | --- |
