# Supplementary material for: Association of Common Variants in TNFRSF13B, TNFSF13, and ANXA3 with Serum Levels of Non-Albumin Protein and Immunoglobulin Isotypes in Japanese
Source: PLoS One. 2012 Apr 27;7(4):e32683. doi: 10.1371/journal.pone.0032683 (PMC3338726; doi:10.1371/journal.pone.0032683)
Supplement: Table S2 — SNPs showed suggestive associations with each examined trait ( P <1.0×10 −6 ). (DOC) [file pone.0032683.s005.doc]

| **Table S2. SNPs showed significant or suggestive associations with each trait (*P* < 1 x 10^-6^)** | | | | | | | | | | |
| --- | --- | --- | --- | --- | --- | --- | --- | --- | --- | --- |
| Trait | SNP | Chr. | Position | A1 | A2 | A2 Freq | Gene | Effect ^a^ | S.E | *P* |
| **TP** | **rs4985726** | 17 | 16804363 | C | G | 0.375 | *TNFRSF13B* | -0.108 | 0.015 | 2.83 x 10^-12^ |
|  | rs4792800 | 17 | 16785892 | A | G | 0.37 | *TNFRSF13B* | -0.106 | 0.015 | 5.38 x 10^-12^ |
|  | rs4561508 | 17 | 16789475 | C | T | 0.371 | *TNFRSF13B* | -0.104 | 0.015 | 6.28 x 10^-12^ |
|  | rs7226097 | 17 | 16809286 | C | T | 0.563 | *TNFRSF13B* | -0.103 | 0.016 | 3.98 x 10^-10^ |
|  | rs4273077 | 17 | 16789864 | A | G | 0.455 | *TNFRSF13B* | -0.092 | 0.015 | 4.34 x 10^-10^ |
|  | rs1052335 | 9 | 6320380 | A | C | 0.129 | *TPD52L3* | -0.112 | 0.022 | 2.86 x 10^-7^ |
|  | rs4985700 | 17 | 16806800 | A | C | 0.261 | *TNFRSF13B* | 0.086 | 0.017 | 4.73 x 10^-7^ |
|  | rs560059 | 9 | 6437196 | A | G | 0.138 | *UHRF2* | -0.112 | 0.022 | 4.89 x 10^-7^ |
|  | rs2280410 | 19 | 54691821 | A | G | 0.845 | *RPS11* | 0.101 | 0.021 | 5.74 x 10**^-^**^7^ |
| **Alb** | **rs1260326** | 2 | 27584444 | C | T | 0.555 | *GCKR* | -0.082 | 0.015 | 3.42 x 10^-8^ |
|  | **rs3817588** | 2 | 27584716 | C | T | 0.698 | *GCKR* | -0.089 | 0.016 | 4.10 x 10^-8^ |
|  | rs780092 | 2 | 27596658 | A | G | 0.299 | *GCKR* | 0.0835 | 0.016 | 2.84 x 10^-7^ |
|  | rs814295 | 2 | 27596719 | A | G | 0.299 | *GCKR* | 0.0832 | 0.016 | 3.11 x 10^-7^ |
| **N-Alb P** | **rs4985726** | 17 | 16804363 | C | G | 0.375 | *TNFRSF13B* | -0.148 | 0.015 | 2.38 x 10^-22^ |
|  | rs4561508 | 17 | 16789475 | C | T | 0.371 | *TNFRSF13B* | -0.143 | 0.015 | 6.36 x 10^-22^ |
|  | rs4792800 | 17 | 16785892 | A | G | 0.37 | *TNFRSF13B* | -0.145 | 0.015 | 8.48 x 10^-22^ |
|  | rs4273077 | 17 | 16789864 | A | G | 0.455 | *TNFRSF13B* | -0.12 | 0.014 | 9.87 x 10^-17^ |
|  | rs7226097 | 17 | 16809286 | C | T | 0.563 | *TNFRSF13B* | -0.12 | 0.016 | 1.34 x 10^-13^ |
|  | **rs3803800** | 17 | 7403693 | A | G | 0.688 | *TNFSF13* | 0.107 | 0.015 | 4.42 x 10^-12^ |
|  | **rs10007186** | 4 | 79808069 | C | T | 0.693 | *ANXA3* | -0.095 | 0.016 | 3.27 x 10^-9^ |
|  | **rs11552708** | 17 | 7403279 | A | G | 0.597 | *TNFSF13* | -0.086 | 0.015 | 3.81 x 10^-9^ |
|  | rs16961828 | 17 | 16760949 | C | G | 0.618 | *TNFRSF13B* | 0.094 | 0.016 | 7.41 x 10^-9^ |
|  | rs4792795 | 17 | 16766741 | A | G | 0.38 | *TNFRSF13B* | -0.09 | 0.016 | 2.24 x 10^-8^ |
|  | rs3751991 | 17 | 16776011 | A | C | 0.616 | *TNFRSF13B* | 0.088 | 0.016 | 2.32 x 10^-8^ |
|  | rs3752005 | 17 | 16769438 | A | T | 0.384 | *TNFRSF13B* | -0.089 | 0.016 | 2.32 x 10^-8^ |
|  | rs11654088 | 17 | 16790538 | C | G | 0.79 | *TNFRSF13B* | -0.1 | 0.019 | 9.93 x 10^-8^ |
|  | rs6739695 | 2 | 100226054 | A | G | 0.587 | *AFF3* | 0.078 | 0.015 | 1.0^1^ x 10^-7^ |
|  | rs10865036 | 2 | 100227027 | C | T | 0.413 | *AFF3* | -0.078 | 0.015 | 1.01 x 10^-7^ |
|  | rs2309811 | 2 | 100228791 | A | G | 0.587 | *AFF3* | 0.078 | 0.015 | 1.01 x 10^-7^ |
|  | rs17017652 | 4 | 91956523 | A | C | 0.606 | *FAM190A* | 0.082 | 0.016 | 1.07 x 10^-7^ |
|  | rs4851274 | 2 | 100237294 | C | T | 0.414 | *AFF3* | -0.078 | 0.015 | 1.07 x 10^-7^ |
|  | rs7597861 | 2 | 100219619 | A | G | 0.413 | *AFF3* | -0.078 | 0.015 | 1.17 x 10^-7^ |
|  | rs6737502 | 2 | 100229335 | C | T | 0.587 | *AFF3* | 0.078 | 0.015 | 1.22 x 10^-7^ |
|  | rs6542921 | 2 | 100230930 | A | C | 0.413 | *AFF3* | -0.078 | 0.015 | 1.26 x 10^-7^ |
|  | rs12474386 | 2 | 100223449 | A | G | 0.417 | *AFF3* | -0.076 | 0.015 | 2.28 x 10^-7^ |
|  | rs9901675 | 17 | 7425536 | A | G | 0.938 | *CD68* | 0.152 | 0.03 | 2.69 x 10^-7^ |
|  | rs2274892 | 17 | 16792752 | G | T | 0.372 | *TNFRSF13B* | 0.074 | 0.015 | 5.61 x 10^-7^ |
|  | rs3818716 | 17 | 16792559 | C | T | 0.628 | *TNFRSF13B* | -0.074 | 0.015 | 5.63 x 10^-7^ |
|  | rs4851273 | 2 | 100214753 | A | G | 0.544 | *AFF3* | 0.072 | 0.014 | 6.46 x 10^-7^ |
|  | rs6542920 | 2 | 100211520 | A | G | 0.544 | *AFF3* | 0.071 | 0.014 | 7.94 x 10^-7^ |
|  | rs893245 | 2 | 100181325 | C | T | 0.539 | *AFF3* | 0.071 | 0.014 | 9.43 x 10^-7^ |
| ^a^ The effect of allele 1 on the normalized values of the indicated trait. | | | | | | | | | | |
| SNPs in bold were selected for replication. | | | | | | | | | | |
| Abbreviations: A1/A2: alleles1 and 2, A2: allele 2, A2 Freq: the frequency of allele 2 estimated by the study, S.E: standard error. | | | | | | | | | | |
