# Supplementary material for: Association of Common Variants in TNFRSF13B, TNFSF13, and ANXA3 with Serum Levels of Non-Albumin Protein and Immunoglobulin Isotypes in Japanese
Source: PLoS One. 2012 Apr 27;7(4):e32683. doi: 10.1371/journal.pone.0032683 (PMC3338726; doi:10.1371/journal.pone.0032683)
Supplement: Table S3 — Haplotype analysis of rs3803800 and rs11552708 in TNFSF13 in association with NAP. (DOC) [file pone.0032683.s006.doc]

| **Table S3. Haplotype analysis of rs3803800 and rs11552708 in association with NAP** | | | | | | | |
| --- | --- | --- | --- | --- | --- | --- | --- |
|  | Haplotype | |  |  |  |  |  |
|  | rs3803800 | rs11552708 |  | Frequency | Effect | S.E | *P* |
| 1 | A | G |  | 0.310 | 0.124 | 0.017 | 2.59 x 10^-13^ |
| 2 | G | G |  | 0.289 | 0.042 | 0.018 | 1.62 x 10^-2^ |
| 3 ^a^ | A | A |  | 0.001 | 0.404 | 0.234 | 8.43 x 10^-2^ |
| 4 ^b^ | G | A |  | 0.399 |  |  |  |
| ^a^ Rare haplotype. | | | | | | | |
| ^b^ The reference haplotype for the analysis. | | | | | | | |
| S.E: standard error. | | | | | | | |
