# Supplementary material for: Association of Common Variants in TNFRSF13B, TNFSF13, and ANXA3 with Serum Levels of Non-Albumin Protein and Immunoglobulin Isotypes in Japanese
Source: PLoS One. 2012 Apr 27;7(4):e32683. doi: 10.1371/journal.pone.0032683 (PMC3338726; doi:10.1371/journal.pone.0032683)
Supplement: Table S4 — Haplotype analysis of rs1260326 and rs3817588 in GCKR in association with ALB. (DOC) [file pone.0032683.s007.doc]

| **Table S4. Haplotype analysis of rs1260326 and rs3817588 in association with ALB** | | | | | | | |
| --- | --- | --- | --- | --- | --- | --- | --- |
|  | Haplotype | |  |  |  |  |  |
|  | rs1260326 | rs3817588 |  | Frequency | Effect | S.E | *P* |
| 1 | C | C |  | 0.293 | -0.101 | 0.017 | 2.83 x 10^-9^ |
| 2 | C | T |  | 0.150 | -0.048 | 0.022 | 2.68 x 10^-2^ |
| 3 ^a^ | T | C |  | 0.007 | -0.033 | 0.093 | 7.24 x 10^-1^ |
| 4 ^b^ | T | T |  | 0.550 |  |  |  |
| ^a^ Rare haplotype. | | | | | | | |
| ^b^ The reference haplotype for the analysis. | | | | | | | |
| S.E: standard error. | | | | | | | |
